# FAK/SRC-JNK axis promotes ferroptosis via upregulating ACSL4 expression

**DOI:** 10.1038/s41419-026-08570-y

**Published:** 2026-03-20

**Authors:** Jianhua Qin, Shuang Ma, Junyang Wang, Siyuan Huang, Jing Luan, Jiyuan He, Guoyuan Hou, Na Sun, Wei Zhang, Minghui Gao

**Affiliations:** 1 The HIT Center for Life Sciences, School of Life Science and Technology, Harbin Institute of Technology, Harbin, China; 2 Department of Microbiology and Immunology, Weill Cornell Medicine, York Avenue, New York, NY USA

**Keywords:** Cell death, Cell signalling

## Abstract

Ferroptosis, an iron-dependent form of programmed cell death driven by toxic lipid peroxide accumulation, plays a critical role in various diseases, making its modulation a promising therapeutic strategy. In this study, we identified defactinib, a specific inhibitor of FAK as a novel ferroptosis suppressors. We demonstrate that FAK/SRC-JNK signaling positively regulates ferroptosis by upregulating ACSL4, a critical mediator of ferroptosis. We reveal that a subset of JNK downstream transcription factors, including ATF2, NFATC1, NFATC3, and SMAD4, promote ferroptosis through direct binding to the ACSL4 promoter and activation of its expression. In contrast, another subset of JNK-associated transcription factors, including c-Jun, STAT3, ELK1, and HSF1, inhibit ferroptosis by binding to the ACSL4 promoter and repressing its expression. The net effect of FAK/SRC-JNK signaling in our models is a significant upregulation of ACSL4 and promotion of ferroptosis. Notably, elevated FAK/SRC-JNK signaling sensitizes cancer cells to ferroptosis-inducing therapies, while inhibition of the FAK/SRC-JNK signaling pathway protects against acute pancreatitis by suppressing ferroptosis. These findings highlight the central role of FAK/ SRC-JNK signaling in controlling ferroptotic cell death and underscore the therapeutic potential of targeting FAK/ SRC-JNK mediated ferroptosis, offering new avenues for the treatment of cancer and acute pancreatitis.

## Introduction

Ferroptosis is a distinct form of programmed cell death characterized by iron-dependent accumulation of lethal lipid peroxides, resulting from the disruption of cellular lipid repair mechanisms. This process is driven by the depletion of intracellular glutathione (GSH) and the inhibition of glutathione peroxidase 4 (GPX4) activity, a key enzyme responsible for reducing lipid peroxides to non-toxic lipid alcohols. When GPX4 activity is compromised, excessive lipid peroxides accumulate, leading to the generation of lethal reactive oxygen species (ROS) and subsequent cell death [1–6]. Central to the regulation of ferroptosis is acyl-CoA synthetase long-chain family member 4 (ACSL4), which facilitates the incorporation of polyunsaturated fatty acids (PUFAs) into membrane lipids, thereby enhancing cellular susceptibility to lipid peroxidation and ferroptotic cell death [7, 8].

Ferroptosis has emerged as a critical player in a wide range of diseases [1, 4, 5]. In cancer, ferroptosis functions as a tumor suppressor mechanism, and its loss of function can promote tumor development and progression. Ferroptosis is crucial for the tumor suppression function of many tumor suppressors, such as p53 [9], Fumarate Hydratase (FH) [10] and BAP1 [11]. Interestingly, cancer cells with specific mutations or metabolic adaptations often exhibit heightened vulnerability to ferroptosis, making it a promising therapeutic target for drug-resistant cancer [12–20]. Beyond oncology, ferroptosis has been implicated in the pathogenesis of various organ injuries, such as heart [21–23], kidney [24], brain [25] et al, where excessive lipid peroxidation contributes to tissue damage and dysfunction. Given its pivotal role in disease, the pharmacological modulation of ferroptosis—through either its induction or inhibition—holds significant therapeutic potential. Inducing ferroptosis could provide a novel strategy for targeting therapy-resistant cancers, while inhibiting ferroptosis may offer protection against organ injuries and other conditions associated with pathological lipid peroxidation.

Focal adhesion kinase (FAK) is an integrin-associated protein tyrosine kinase that is frequently overexpressed in advanced human cancers [26, 27]. SRC is a membrane-anchored tyrosine kinase, which mediates signaling induced by a wide range of cell surface receptors, leading to cell growth and adhesion [28]. Similar to FAK, overexpression or activation of SRC is also frequently observed in a large number of human malignancies [29]. Activation of FAK/SRC signal leads to activation of c-Jun N-terminal kinase (JNK), a stress-activated protein kinase involved in diverse cellular processes, including apoptosis, autophagy, and inflammation [30]. While the role of this signaling axis in promoting tumorigenesis and therapy resistance is well-documented, its involvement in regulating ferroptosis has only recently begun to be explored.

In this study, we identified that FAK/SRC-JNK signaling positively modulates ferroptosis through the upregulation of ACSL4, a key regulator of ferroptosis. We further elucidated that multiple transcription factors downstream of JNK signaling, including ATF2, NFATC1, NFATC3, and SMAD4, enhance ferroptosis by binding to the promoter region of ACSL4 and driving its expression. Conversely, Four JNK downstream transcription factors—c-Jun, ELK1, STAT3 and HSF1—were found to negatively regulate ferroptosis by directly binding to the ACSL4 promoter and suppressing its expression. Inhibition of the FAK/SRC-JNK signaling pathway protects against acute pancreatitis by suppressing ferroptosis, while elevated FAK/SRC-JNK levels sensitized cancer cells to ferroptosis-inducing treatments in a mouse xenograft model. These findings underscore the pivotal role of FAK/SRC-JNK signaling in regulating ferroptotic cell death and suggest that mutations within this pathway may serve as predictive biomarkers for the responsiveness of cancer cells to ferroptosis-inducing therapies.

## Results

### FAK/Src complex positively regulate ferroptosis

**Fig. 1:**
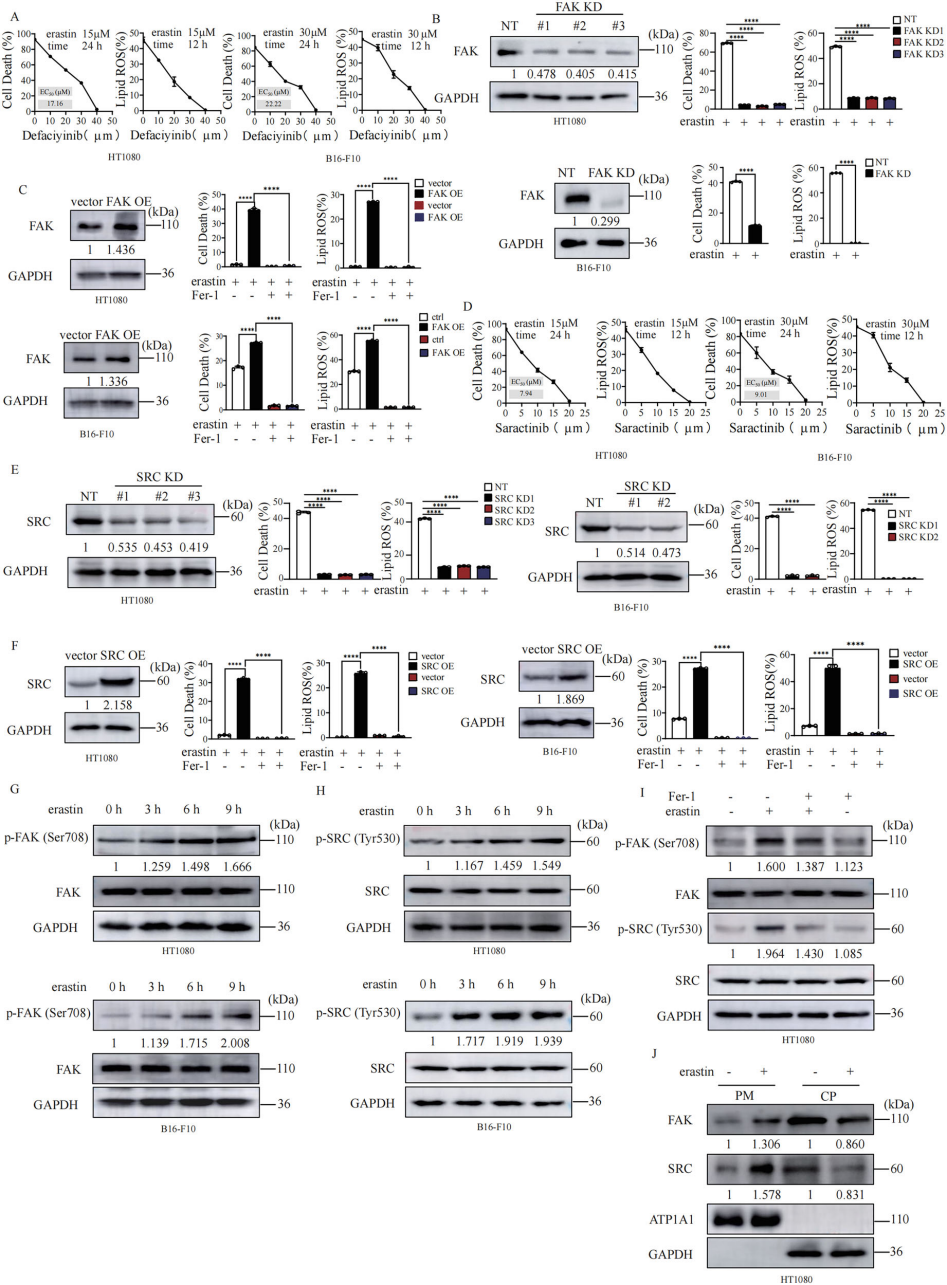
FAK/SRC complex positively regulate ferroptosis. **A** FAK inhibitor defactinib treatment suppresses erastin induced cell death and lipid peroxidation. Cells as indicated were treated with erastin (15 μM for HT1080 cells and 30 μM for B16-F10 cells) with or without defactinib for 24 h to measure cell death, or 12 h to measure lipid peroxidation. Cells were stained with Propidium Iodide (PI) and cell death was analyzed by flow cytometry. Cells treated as indicated were stained with BODIPY 581/591 C11, and lipid ROS levels were measured by flow cytometry. **B** Knockdown of FAK suppresses erastin induced cell death and lipid peroxidation. Cells as indicated were treated with erastin (15 μM for HT1080 cells and 30 μM for B16-F10 cells) for 14 h, then cell death and lipid ROS were measured as described in Fig.1A. Western blot images confirm the expression of the indicated protein. **C** Overexpression of FAK promotes erastin induced cell death and lipid peroxidation. Cells as indicated were treated with erastin (10 μM for HT1080 cells and 30 μM for B16-F10 cells) for 14 h for cell death measurement, or 12 h for lipid ROS measurement. Cell death and lipid ROS were measured as described in Fig.1A. Western blot images confirm the expression of the indicated protein. **D** SRC inhibitor saracatinib treatment suppresses erastin induced cell death and lipid peroxidation. Cells as indicated were treated with erastin (10 μM for HT1080 cells and 30 μM for B16-F10 cells) with or without saracatinib (40 μM) for 14 h for cell death measurement, or 12 h for lipid ROS measurement. Cell death and lipid ROS were measured as described in Fig.1A. **E** Knockdown of SRC suppresses erastin induced cell death and lipid peroxidation. Cells as indicated were treated with erastin as in Fig.1D, then cell death and lipid ROS were measured as described in Fig.1A. Western blot images confirm the expression of the indicated protein. **F** Overexpression of SRC promotes erastin induced cell death and lipid peroxidation. Cells as indicated were treated with erastin (10 μM for HT1080 cells and 30 μM for B16-F10 cells) for 12 h for cell death measurement, or 10 h for lipid ROS measurement. Cell death and lipid ROS were measured as described in Fig.1A. Western blot images confirm the expression of the indicated protein. **G** Erastin treatment induced time dependent phosphorylation of FAK. Cells as indicated were treated with erastin (10 μM for HT1080 cells and 30 μM for B16-F10 cells) for indicated time, western blot images confirm the expression of the indicated protein. **H** Erastin treatment induced time dependent phosphorylation of SRC. Cells as indicated were treated with erastin (10 μM for HT1080 cells and 30 μM for B16-F10 cells) for indicated time, western blot images confirm the expression of the indicated protein. **I** Ferrostin-1 treatment blocked erastin induced phosphorylation of FAK and SRC. HT1080 cells were treated with erastin (10 μM) with or without Fer-1 (0.2 μM) for indicated time, western blot images confirm the expression of the indicated protein. **J** Erastin treatment induced cell membrane translocation of FAK and SRC. HT1080 cells were treated with erastin (10 μM) for 9 h. Western blot analysis of the cell membrane accumulation of FAK and SRC. GAPDH: the biomarker of cytoplasm (CP), ATP1A1: the biomarker of membrane. Data are derived from three independent experiments, and each value represents the mean ± SD. *P < 0.05, **P < 0.01, ***P < *0.001*, ****P < *0.0001*, *t* test. (*n* = 3).

To identify potent compounds that modulate ferroptosis, we screened a bioactive compounds library with 4,417 compounds using erastin-induced MEFs ferroptosis as an assay (Fig. S1A). Many reported ferroptosis inhibitors were identified in our screening, we also identified that defactinib, a specific inhibitor of FAK significantly suppresses erastin induced lipid peroxidation and ferroptosis in both HT1080 human fibrosarcoma cells and B16 mouse melanoma cells in dose-dependent manner (Fig. 1A). To verify the role of FAK in ferroptosis, we created FAK knocking down (FAK KD) cells by using three different short hairpin RNAs. We found that depletion of FAK significantly blocked erastin induced lipid peroxidation and ferroptosis in both HT1080 and B16-F10 cells (Fig. 1B), and reconstitution of FAK back to FAK KD cells restores ferroptosis sensitivity both HT1080 and B16-F10 cells (Fig. S1B). Consistently, overexpression of FAK in both HT1080 cells and B16-F10 cells (Fig. 1C) promotes lipid peroxidation and ferroptosis. These results indicating that FAK is a positive regulator of ferroptosis.

FAK and SRC are intracellular (nonreceptor) tyrosine kinases that physically and functionally interact to promote a variety of cellular responses. We therefore tested the role of SRC in ferroptosis. Saracatinib, a specific inhibitor of SRC suppresses erastin induced lipid peroxidation and ferroptosis in both HT1080 cells and B16 cells in a dose dependent manner (Fig. 1D). Knockdown of SRC significantly blocked erastin induced lipid peroxidation and ferroptosis in both HT1080 and B16-F10 cells (Fig. 1E), and reconstitution of c-Src back to SRC KD cells restores ferroptosis sensitivity in both HT1080 cell and B16-F10 cells (Fig. S1C). Furthermore, overexpression of SRC sensitize cells to lipid peroxidation and ferroptosis in both HT1080 and B16-F10 cells (Fig. 1F). More importantly, we found that treatment with ferroptosis inducer erastin resulted in time dependent phosphorylation of FAK (Fig. 1G) and SRC (Fig. 1H) which is partially blocked by ferroptosis inhibitor ferrostatin-1 (Fig. 1I) and let to cell membrane translocation of FAK and SRC (Fig. 1J). RSL3 induces ferroptosis by directly inhibiting the enzyme activity of GPX4. We found that knocking down of FAK or SRC could also block RSL3 induced accumulation of lipid ROS and ferroptosis (Fig. S1D, E). These data suggest that FAK/SRC complex are activated upon ferroptosis induction and FAK/SRC complex positively regulate ferroptosis.

### FAK/SRC promotes ferroptosis by upregulation of ACSL4

**Fig. 2:**
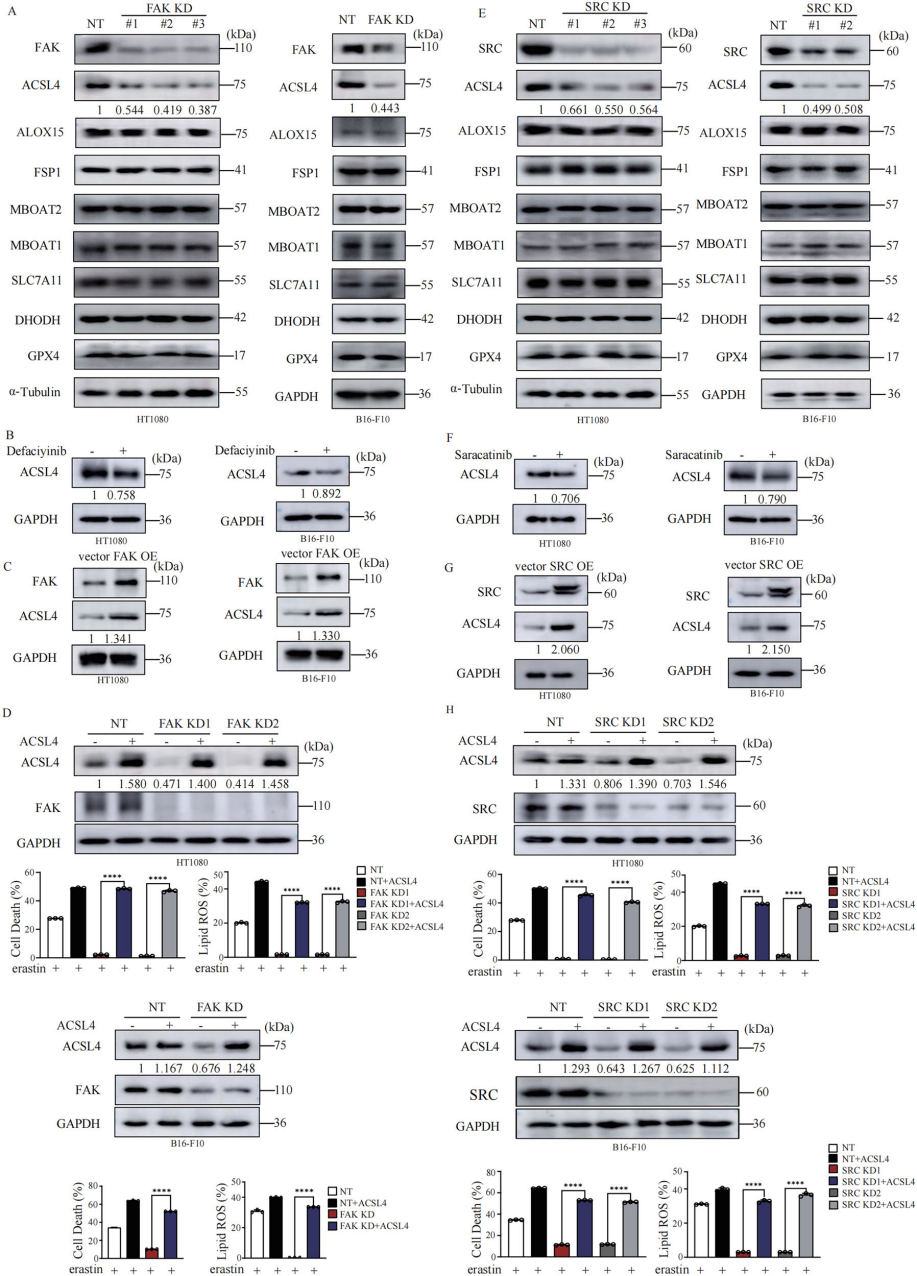
FAK/SRC promotes ferroptosis by upregulation of ACSL4. **A** Knocking down of FAK results in downregulation of ACSL4 expression in HT1080 and B16-F10 cells. Western blot images confirm the expression of the indicated proteins. **B** FAK inhibitor defactinib treatment suppresses the expression of ACSL4 in HT1080 and B16-F10 cells. Western blot images confirm the expression of the indicated proteins. defactinib (20 μM for 12 h). **C** Overexpression of FAK promotes the expression of ACSL4 in HT1080 cells and B16-F10 cells. Western blot images confirm the expression of the indicated proteins. **D** Overexpression of ACSL4 restores the sensitivity to erastin induced lipid peroxidation and ferroptosis of FAK KD cells. Western blot images confirm the expression of the indicated proteins in indicated cells. Cells as indicated were treated with erastin (10 μM for HT1080 cells and 30 μM for B16-F10 cells) for 14 h for cell death measurement, or 12 h for lipid ROS measurement, cell death and lipid ROS were measured as described in Fig. 1A. **E** Knocking down of SRC results in downregulation of ACSL4 expression in HT1080 and B16-F10 cells. Western blot images confirm the expression of the indicated proteins. **F** SRC inhibitor saracatinib treatment suppresses the expression of ACSL4 in HT1080 and B16-F10 cells. Western blot images confirm the expression of the indicated proteins. saracatinib (40 μM for 12 h). **G** Overexpression of SRC promotes the expression of ACSL4 in HT1080 cells and B16-F10 cells. Western blot images confirm the expression of the indicated proteins. **H** Overexpression of ACSL4 restores the sensitivity to erastin induced lipid peroxidation and ferroptosis of SRC KD cells. Western blot images confirm the expression of the indicated proteins in indicated cells. Cells as indicated were treated with erastin (10 μM for HT1080 cells and 30 μM for B16-F10 cells) for 14 h for cell death measurement, or 12 h for lipid ROS measurement, cell death and lipid ROS were measured as described in Fig. 1A. Data are derived from three independent experiments, and each value represents the mean ± SD. *P < 0.05, **P < 0.01, ***P < 0.001, ****P < *0.0001*, *t* test. (*n* = 3).

Next, we sought to determine how FAK/SRC regulates ferroptosis sensitivity. We compared the expression level of multiple well-studied regulators of ferroptosis between control cells and FAK KD cells. We observed that only the expression of ACSL4, a crucial positive regulator of ferroptosis, decreased in both FAK KD HT1080 and B16-F10 cells (Fig. 2A). Knocking down of FAK also decreases the mRNA expression of ACSL4 in HT1080 and B16-F10 cells (Fig. S2A). Consistently, treatment with defactinib, FAK inhibitor, inhibits ACSL4 expression in both HT1080 and B16-F10 cells on both protein level (Fig. 2B) and mRNA level (Fig. S2B). Reconstitution of FAK back to FAK KD cells restores the expression level of ACSL4 (Fig. S2C). Overexpression of FAK promotes ACSL4 expression in both HT1080 and B16-F10 cells on both protein level (Fig. 2C) and mRNA level (Fig. S2D). More importantly, reconstitution of ACSL4 into FAK KD cells completely rescued the sensitivity to lipid peroxidation and ferroptosis in both HT1080 and B16-F10 cells (Fig. 2D). Similarly, genetic knocking down or pharmaceutical inhibition of SRC specifically suppress the expression of ACSL4 in both HT1080 and B16-F10 cells on both protein level (Fig. 2E, F) and mRNA level (Fig. S2E, F). Reconstitution of SRC back to SRC KD cells restore the expression level of ACSL4 in both HT1080 and B16-F10 cells (Fig. S2G). Overexpression of SRC promotes ACSL4 expression in both HT1080 and B16-F10 cells on both protein level (Fig. 2G) and mRNA level (Fig. S2H). Reconstitution of ACSL4 back to SRC KD HT1080 cells rescued the sensitivity to lipid peroxidation and ferroptosis (Fig. 2H). These data suggest that FAK/SRC promotes ferroptosis by upregulation of ACSL4.

### JNK1/2 positively regulates ferroptosis via upregulation of ACSL4

**Fig. 3:**
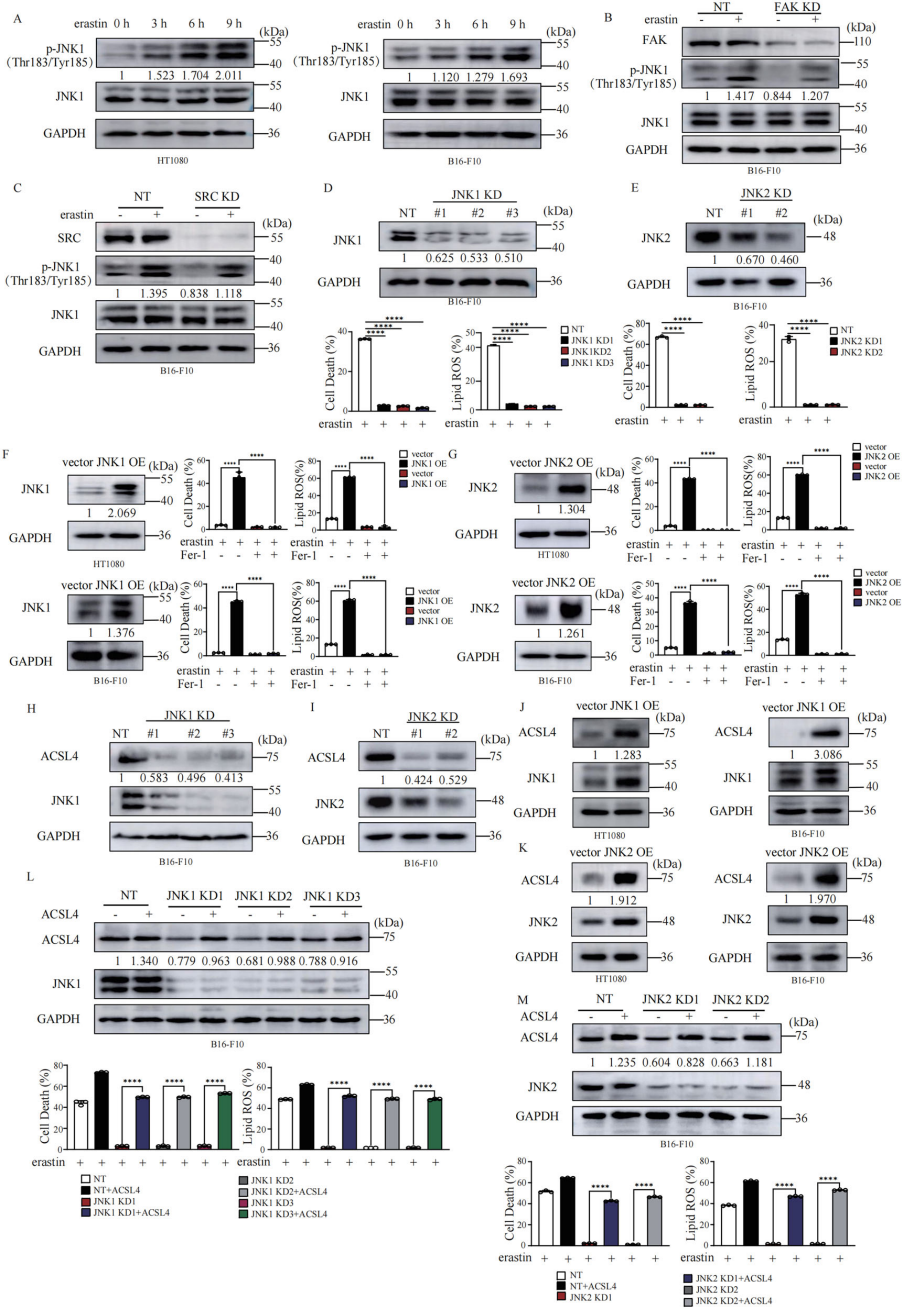
JNK1/2 positively regulates ferroptosis via upregulation of ACSL4. **A** Erastin treatment induced time dependent phosphorylation of JNK. Cells as indicated were treated with erastin (10 μM for HT1080 cells and 30 μM for B16-F10 cells) for indicated time, western blot images confirm the expression of the indicated protein. **B** Knocking down of FAK decreases erastin induced phosphorylation of JNK1. B16-F10 cells as indicated were treated with erastin (30 μM) for 9 h, western blot images confirm the expression of the indicated protein. **C** Knocking down of SRC decreases erastin induced phosphorylation of JNK1. B16-F10 cells as indicated were treated with erastin (30 μM) for 9 h, western blot images confirm the expression of the indicated protein. **D** Knocking down of JNK1 blocks erastin inducd ferroptosis and lipid peroxidation. B16-F10 cells as indicated were treated with erastin (30 μM) for 14 h for cell death measurement, or 12 h for lipid ROS measurement. Cell death and lipid ROS were measured as described in Fig. 1A. Western blot images confirm the expression of the indicated protein. **E** Knocking down of JNK2 blocks erastin inducd ferroptosis and lipid peroxidation. B16-F10 cells as indicated were treated with erastin (30 μM) for 14 h for cell death measurement, or 12 h for lipid ROS measurement. Cell death and lipid ROS were measured as described in Fig. 1A. Western blot images confirm the expression of the indicated protein. **F** Overexpression of JNK1 promotes ferroptosis and lipid peroxidation in HT1080 and B16-F10 cells. Cells as indicated were treated with erastin (10 μM for HT1080 cells and 30 μM for B16-F10 cells) for 12 h for cell death measurement, or 10 h for lipid ROS measurement. Cell death and lipid ROS were measured as described in Fig. 1A. Western blot images confirm the expression of the indicated protein. **G** Overexpression of JNK2 promotes ferroptosis and lipid peroxidation in HT1080 and B16-F10 cells. Cells as indicated were treated with erastin (10 μM for HT1080 cells and 30 μM for B16-F10 cells) for 12 h for cell death measurement, or 10 h for lipid ROS measurement. Cell death and lipid ROS were measured as described in Fig. 1A. Western blot images confirm the expression of the indicated protein. **H** Knocking down of JNK1 resultes in downregulation of ACSL4 expression in HT1080. Western blot images confirm the expression of the indicated protein. **I** Knocking down of JNK1 resultes in downregulation of ACSL4 expression in B16-F10 cells. Western blot images confirm the expression of the indicated protein. **J** Overexpression of JNK1 promotes the expression of ACSL4 in HT1080 cells and B16-F10 cells. Western blot images confirm the expression of the indicated proteins. **K** Overexpression of JNK2 promoted the expression of ACSL4 in HT1080 cells and B16-F10 cells. Western blot images confirm the expression of the indicated proteins. **L** Overexpression of ACSL4 restores the sensitivity to erastin induced lipid peroxidation and ferroptosis of JNK1 KD B16-F10 cells. Western blot images confirm the expression of the indicated proteins in indicated cells. Cells as indicated were treated with erastin (30 μM) for 14 h for cell death measurement, or 12 h for lipid ROS measurement. Cell death and lipid ROS were measured as described in Fig. 1A. **M** Overexpression of ACSL4 restores the sensitivity to erastin induced lipid peroxidation and ferroptosis of JNK2 KD B16-F10 cells. Western blot images confirm the expression of the indicated proteins in indicated cells. Cells as indicated were treated with erastin (30 μM) for 14 h for cell death measurement, or 12 h for lipid ROS measurement. Cell death and lipid ROS were measured as described in Fig. 1A. Data are derived from three independent experiments, and each value represents the mean ± SD. *P < 0.05, **P < 0.01, ***P < *0.001*, ****P < *0.0001, t* test. (*n* = 3).

JNK is one of the major downstream factors of FAK/SRC. There are three JNK coding genes. Whereas, JNK1 and JNK2 have a broad tissue distribution, JNK3 is mainly localized in neurons and to a lesser extent in the heart and the testis [31]. We therefore focused on the role of JNK1 and JNK2 on ferroptosis. As shown in Fig. 3A, treatment with erastin induces time-dependent phosphorylation of JNK1 in both HT1080 and B16-F10 cells, suggesting JNK1 is activated during ferroptosis. The phosphorylation of JNK1 upon erastin is partially dependent on FAK/SRC complex (Fig. 3B, C). Knockdown of JNK1 or JNK2 dramatically suppresses lipid peroxidation and ferroptosis (Fig. 3D, E). And overexpression of JNK1 or JNK2 enhances the ferroptosis sensitivity (Fig. 3F, G). More importantly, knockdown of JNK1 or JNK2 dramatically decreased the expression of ACSL4 on both protein level (Fig. 3H, I) and mRNA level (Fig. S2A). Reconstitution of JNK1 back to JNK1 KD cells or reconstitution of JNK2 back to JNK2 KD cells restored the ferroptosis sensitivity and expression level of ACSL4 (Fig. S2B, C). Furthermore, overexpression of JNK1 or JNK2 promoted the expression of ACSL4 on both protein level (Fig. 3J, K) and mRNA level (Fig. S2D). Reconstitution of ACSL4 back to JNK1 KD cells or JNK2 KD cells completely restore the sensitivity to lipid peroxidation and ferroptosis (Fig. 3L, M). Furthermore, we also analyzed whether JNK1 regulates the expression of other ferroptosis regulators, such as GPX4 or NCOA4 mediated ferritin turnover. As shown in Fig. S3E, overexpression of JNK1 does not change the protein level of these key nodes of ferroptosis. Our data, where knocking down either one was sufficient to significantly suppress ferroptosis and ACSL4 expression, suggest a redundant or partially redundant role for JNK1 and JNK2 in this specific ferroptosis regulatory axis. We further generated JNK1/JNK2 double knockdown cells and determined their ACSL4 expression and ferroptosis sensitivity. We found that double knocking down of JNK1 and JNK2 further suppresses the expression of ACSL4 and ferroptosis sensitivity compared to JNK1 or JNK2 knockdown alone (Fig. S3F), further supporting the idea that both isoforms contribute to the full regulatory effect. Consistent to FAK and SRC, knockdown of JNK1 significantly suppressed RSL3-induced lipid peroxidation and cell death (Fig. S3G). Above data indicate that regulation of ferroptosis by JNK is dependent on ACSL4.

### Regulation of ferroptosis by JNK downstream transcriptional factors

**Fig. 4:**
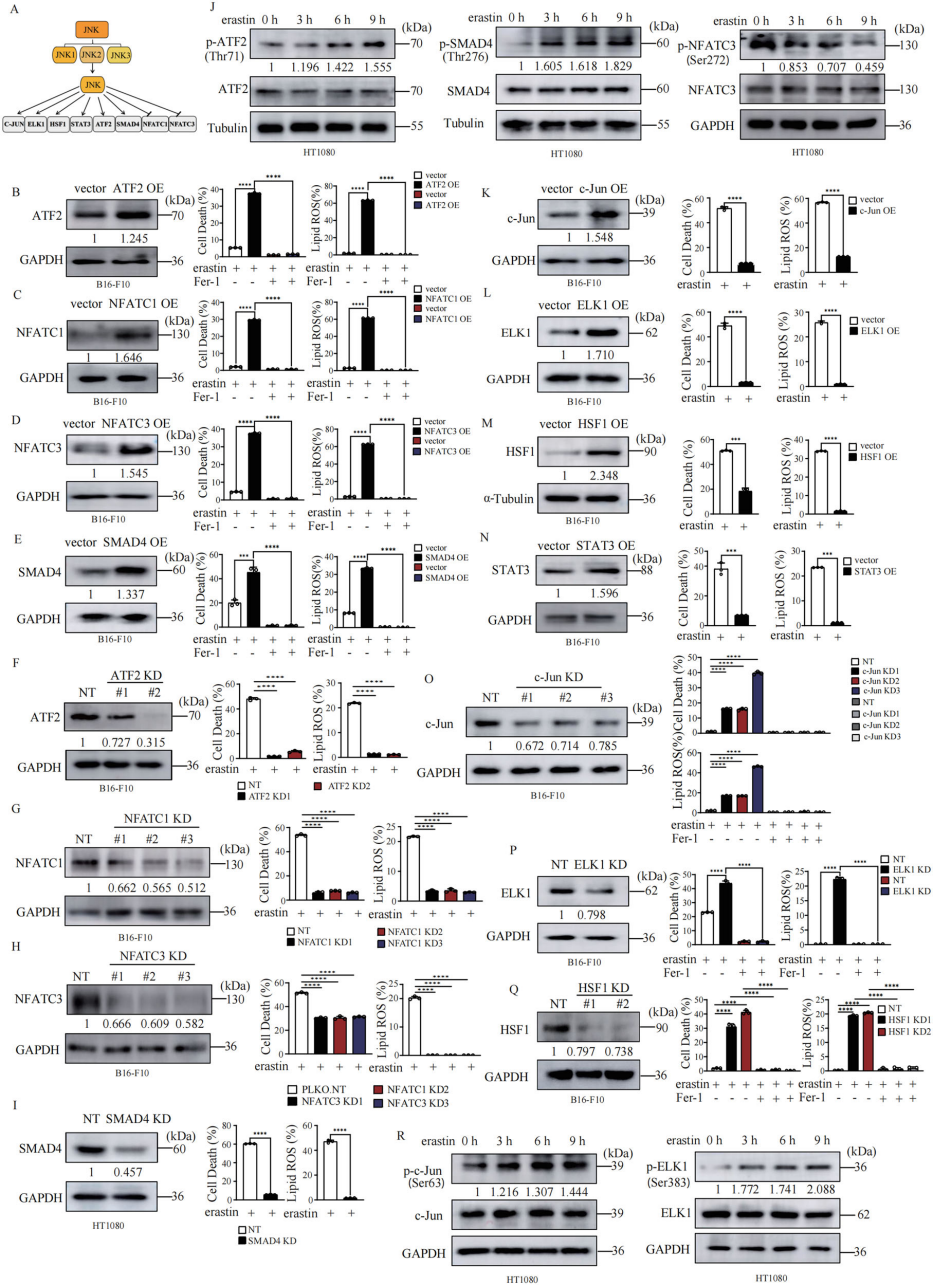
JNK downstream transcriptional factors regulate cells susceptibility to ferroptosis. **A** Schematic diagram of JNK downstream transcriptional factors. **B** Overexpression of ATF2 promotes ferroptosis. B16-F10 cells as indicated were treated with erastin (30 μM) for 14 h for cell death measurement, or 12 h for lipid ROS measurement. Cell death and lipid ROS were measured as described in Fig. 1A. Western blot images confirm the expression of the indicated proteins in indicated cells. **C** Overexpression of NFATC1 promotes ferroptosis. B16-F10 cells as indicated were treated with erastin (30 μM) for 14 h for cell death measurement, or 12 h for lipid ROS measurement. Cell death and lipid ROS were measured as described in Fig. 1A. Western blot images confirm the expression of the indicated proteins in indicated cells. **D** Overexpression of NFATC3 promotes ferroptosis. B16-F10 cells as indicated were treated with erastin (30 μM) for 14 h for cell death measurement, or 12 h for lipid ROS measurement. Cell death and lipid ROS were measured as described in Fig. 1A. Western blot images confirm the expression of the indicated proteins in indicated cells. **E** Overexpression of SMAD4 promotes ferroptosis. B16-F10 cells as indicated were treated with erastin (30 μM) for 14 h for cell death measurement, or 12 h for lipid ROS measurement. Cell death and lipid ROS were measured as described in Fig. 1A. Western blot images confirm the expression of the indicated proteins in indicated cells. **F** Knockdown of ATF2 suppresses ferroptosis. B16-F10 cells as indicated were treated with erastin (30 μM) for 14 h for cell death measurement, or 12 h for lipid ROS measurement. Cell death and lipid ROS were measured as described in Fig. 1A. Western blot images confirm the expression of the indicated proteins in indicated cells. **G** Knockdown of NFATC1 suppresses ferroptosis. B16-F10 cells as indicated were treated with erastin (30 μM) for 14 h for cell death measurement, or 12 h for lipid ROS measurement. Cell death and lipid ROS were measured as described in Fig. 1A. Western blot images confirm the expression of the indicated proteins in indicated cells. **H** Knockdown of NFATC3 suppresses ferroptosis. B16-F10 cells as indicated were treated with erastin (30 μM) for 14 h for cell death measurement, or 12 h for lipid ROS measurement. Cell death and lipid ROS were measured as described in Fig. 1A. Western blot images confirm the expression of the indicated proteins in indicated cells. **I** Knockdown of SMAD4 suppresses ferroptosis. B16-F10 cells as indicated were treated with erastin (30 μM) for 14 h for cell death measurement, or 12 h for lipid ROS measurement. Cell death and lipid ROS were measured as described in Fig. 1A. Western blot images confirm the expression of the indicated proteins in indicated cells. **J** Erastin treatement promoted phosphorylation of ATF2, SMAD4, but suppressed phosphorylation of NFATC3 in HT1080 cells. HT1080 cells were treated with erastin (10 μM) as indicated, western blot images confirm the expression of the indicated proteins. **K** Overexpression of c-Jun suppresses ferroptosis. B16-F10 cells as indicated were treated with erastin (30 μM) for 14 h for cell death measurement, or 12 h for lipid ROS measurement. Cell death and lipid ROS were measured as described in Fig. 1A. Western blot images confirm the expression of the indicated proteins in indicated cells. **L** Overexpression of ELK1 suppresses ferroptosis. B16-F10 cells as indicated were treated with erastin (30 μM) for 14 h for cell death measurement, or 12 h for lipid ROS measurement. Cell death and lipid ROS were measured as described in Fig. 1A. Western blot images confirm the expression of the indicated proteins in indicated cells. **M** Overexpression of HSF1 suppresses ferroptosis. B16-F10 cells as indicated were treated with erastin (30 μM) for 14 h for cell death measurement, or 12 h for lipid ROS measurement. Cell death and lipid ROS were measured as described in Fig. 1A. Western blot images confirm the expression of the indicated proteins in indicated cells. **N** Overexpression of STAT3 suppresses ferroptosis. B16-F10 cells as indicated were treated with erastin (30 μM) for 14 h for cell death measurement, or 12 h for lipid ROS measurement. Cell death and lipid ROS were measured as described in Fig. 1A. Western blot images confirm the expression of the indicated proteins in indicated cells. **O** Knockdown of c-Jun promotes ferroptosis. B16-F10 cells as indicated were treated with erastin (30 μM) for 14 h for cell death measurement, or 12 h for lipid ROS measurement. Cell death and lipid ROS were measured as described in Fig. 1A. Western blot images confirm the expression of the indicated proteins in indicated cells. **P** Knockdown of ELK1 promotes ferroptosis. B16-F10 cells as indicated were treated with erastin (30 μM) for 14 h for cell death measurement, or 12 h for lipid ROS measurement. Cell death and lipid ROS were measured as described in Fig. 1A. Western blot images confirm the expression of the indicated proteins in indicated cells. **Q** Knockdown of HSF1 promotes ferroptosis. B16-F10 cells as indicated were treated with erastin (30 μM) for 14 h for cell death measurement, or 12 h for lipid ROS measurement. Cell death and lipid ROS were measured as described in Fig. 1A. Western blot images confirm the expression of the indicated proteins in indicated cells. **R** Erastin treatment induced time dependent phosphorylation of c-Jun and ELK1. HT1080 cells were treated with erastin (10 μM) as indicated, western blot images confirm the expression of the indicated proteins. Data are derived from three independent experiments, and each value represents the mean ± SD. *P < 0.05, **P < 0.01, ***P < *0.001*, ****P < *0.0001*, *t* test. (*n* = 3).

JNK controls cell fate determination through a serial of transcriptional factors [30], including ATF2 [32], NFATC1 [33], NFATC3 [33], SMAD4 [34], c-Jun [35], ELK1 [36], HSF1 [37], and STAT3 [38] (Fig. 4A). We then tried to determine the function of these transcriptional factors in ferroptosis. As shown in Fig. 4B–E, overexpression of ATF2 (Fig. 4B), NFATC1 (Fig. 4C), NFATC3 (Fig. 4D), and SMAD4 (Fig. 4E) individually promoted lipid peroxidation and ferroptosis. Knockdown of ATF2 (Fig. 4F), NFATC1 (Fig. 4G), NFATC3 (Fig. 4H), and SMAD4 (Fig. 4I) individually significantly suppressed lipid peroxidation and ferroptosis. Furthermore, activation of SMAD4 by kartogenin (KGN) significantly enhanced erastin increased lipid peroxidation and ferroptosis in both HT1080 and B16-F10 cells (Fig. S4A), and inhibition of NFATC3 by NDMC101 significantly suppressed erastin induced lipid peroxidation and ferroptosis in both HT1080 and B16-F10 cells (Fig. S4B). Additionally, we found that both ATF2 and SMAD4 are phosphorylated in a time-dependent manner upon erastin treatment and NFATC3 is dephosphorylated in a time-dependent manner upon erastin treatment (Fig. 4J).

Unlike the above transcription factors, overexpression of c-Jun (Fig. 4K), ELK1 (Fig. 4L), HSF1 (Fig. 4M), and STAT3 (Fig. 4N) individually in B16-F10 cells suppressed lipid peroxidation and ferroptosis. Knockdown of c-Jun (Fig. 4O), ELK1 (Fig. 4P) and HSF1 (Fig. 4Q) individually in B16-F10 cells significantly promoted lipid peroxidation and ferroptosis. Furthermore, inhibition of STAT3 by STAT3-IN-1 significantly promoted erastin induced lipid peroxidation and ferroptosis in both HT1080 and B16-F10 cells (Fig. S4C). Inhibition of HSF1 by DTHIB significantly promoted erastin induced lipid peroxidation and ferroptosis in both HT1080 and B16-F10 cells (Fig. S4D). Activation of HSF1 by HSF1A significantly suppressed erastin induced lipid peroxidation and ferroptosis in HT1080 cells (Fig. S4E). We also observed that ELK1 and c-Jun are phosphorylated in a time-dependent manner upon erastin treatment (Fig. 4R).

### The FAK/SRC -JNK axis promotes ferroptosis via ATF2, NFATC1, NFATC3, and SMAD4 mediated ACSL4 upregulation

**Fig. 5:**
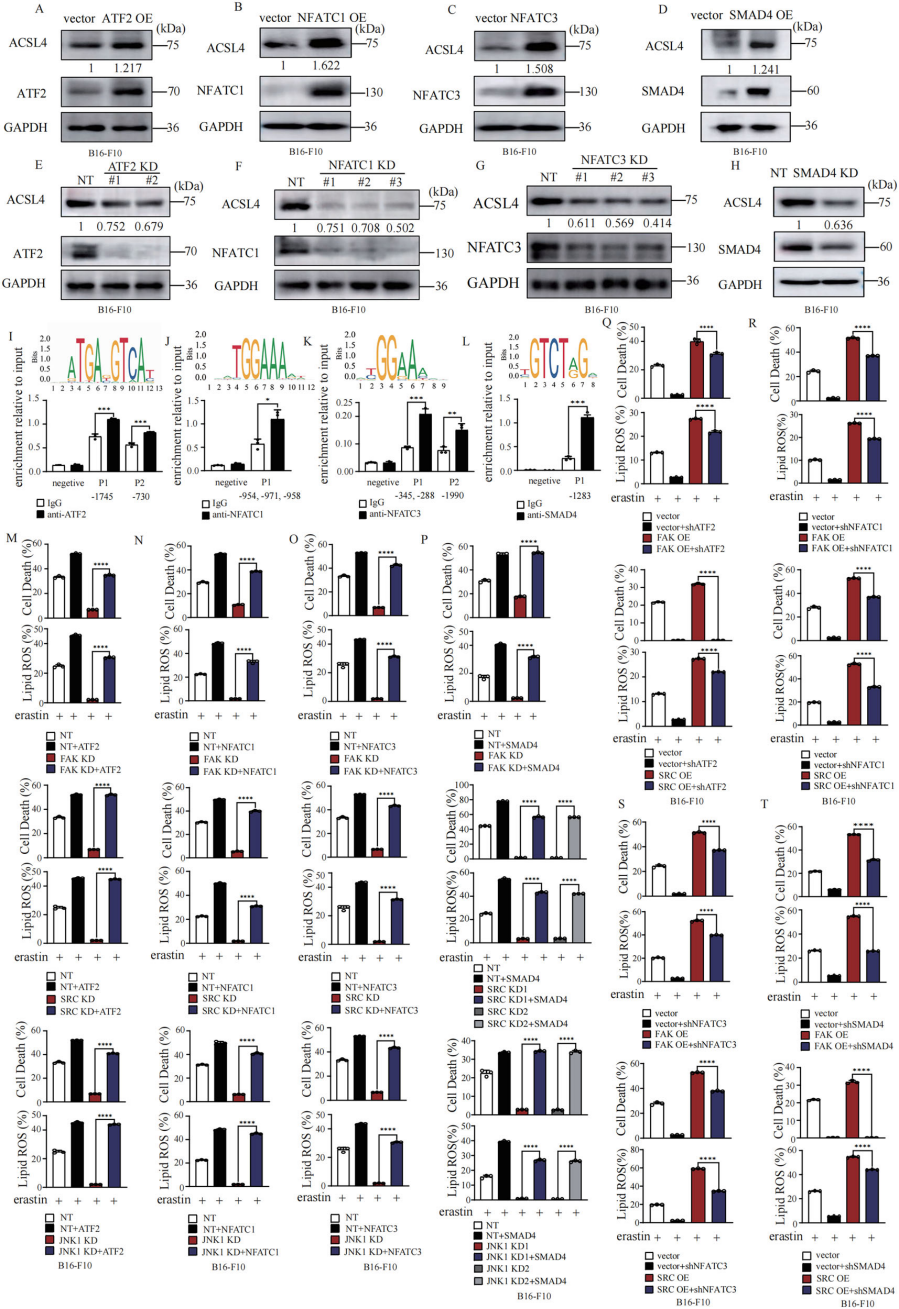
The FAK/SRC-JNK axis promotes ferroptosis via ATF2, SMAD4, NFATC1, and NFATC3, mediated ACSL4 upregulation. **A** Overexpression of ATF2 promotes the expression of ACSL4 in B16-F10 cells. Western blot images confirm the expression of the indicated proteins. **B** Overexpression of NFATC1 promotes the expression of ACSL4 in B16-F10 cells. Western blot images confirm the expression of the indicated proteins. **C** Overexpression of NFATC3 promotes the expression of ACSL4 in B16-F10 cells. Western blot images confirm the expression of the indicated proteins. **D** Overexpression of SMAD4 promotes the expression of ACSL4 in B16-F10 cells. Western blot images confirm the expression of the indicated proteins. **E** Knockdown of ATF2 suppresses the expression of ACSL4 in B16-F10 cells. Western blot images confirm the expression of the indicated proteins. **F** Knockdown of NFATC1 suppresses the expression of ACSL4 in B16-F10 cells. Western blot images confirm the expression of the indicated proteins. **G** Knockdown of NFATC3 suppresses the expression of ACSL4 in B16-F10 cells. Western blot images confirm the expression of the indicated proteins. **H** Knockdown of SMAD4 suppresses the expression of ACSL4 in B16-F10 cells. Western blot images confirm the expression of the indicated proteins. **I** ATF2 binds to the promoter region of ACSL4 in HT1080 cells. Chromatin-IP was performed using ATF2 antibody or control IgG. Values are percentage of input. Enrichment was normalized to the IgG control. The binding position on ACSL4 promoter and predicated binding sequence are shown as labelled. **J** NFATC1 binds to the promoter region of ACSL4 in B16-F10 cells. Chromatin-IP was performed using NFATC1 antibody or control IgG. Values are percentage of input. Enrichment was normalized to the IgG control. The binding position on ACSL4 promoter and predicated binding sequence are shown as labelled. **K** NFATC3 binds to the promoter region of ACSL4 in HT1080 cells. Chromatin-IP was performed using NFATC3 antibody or control IgG. Values are percentage of input. Enrichment was normalized to the IgG control. The binding position on ACSL4 promoter and predicated binding sequence are shown as labelled. **L** SMAD4 binds to the promoter region of ACSL4 in HT1080 cells. Chromatin-IP was performed using SMAD4 antibody or control IgG. Values are percentage of input. Enrichment was normalized to the IgG control. The binding position on ACSL4 promoter and predicated binding sequence are shown as labelled. **M** Overexpression of ATF2 rescues erastin induced ferroptosis and lipid peroxidation of FAK KD, SRC KD or JNK1 KD B16-F10 cells. Cells as indicated were treated with erastin (30 μM) for 14 h for cell death measurement, or 12 h for lipid ROS measurement. Cell death and lipid ROS were measured as described in Fig. 1A. **N** Overexpression of NFATC1 rescues erastin induced ferroptosis and lipid peroxidation of FAK KD, SRC KD or JNK1 KD B16-F10 cells. Cells as indicated were treated with erastin (30 μM) for 14 h for cell death measurement, or 12 h for lipid ROS measurement. Cell death and lipid ROS were measured as described in Fig. 1A. **O** Overexpression of NFATC3 rescues erastin induced ferroptosis and lipid peroxidation of FAK KD, SRC KD or JNK1 KD B16-F10 cells. Cells as indicated were treated with erastin (30 μM) for 14 h for cell death measurement, or 12 h for lipid ROS measurement. Cell death and lipid ROS were measured as described in Fig. 1A. **P** Overexpression of SMAD4 rescues erastin induced ferroptosis and lipid peroxidation of FAK KD, SRC KD or JNK1 KD B16-F10 cells. Cells as indicated were treated with erastin (30 μM) for 14 h for cell death measurement, or 12 h for lipid ROS measurement. Cell death and lipid ROS were measured as described in Fig. 1A. **Q** Knockdown of ATF2 partially suppresses the elevated ferroptosis sensitivity and lipid peroxidation of FAK OE or SRC OE B16-F10 cells. Cells as indicated were treated with erastin (30 μM) for 14 h for cell death measurement, or 12 h for lipid ROS measurement. Cell death and lipid ROS were measured as described in Fig. 1A. **R** Knockdown of NFATC1 partially suppresses the elevated ferroptosis sensitivity and lipid peroxidation of FAK OE or SRC OE B16-F10 cells. Cells as indicated were treated with erastin (30 μM) for 14 h for cell death measurement, or 12 h for lipid ROS measurement. Cell death and lipid ROS were measured as described in Fig. 1A. **S** Knockdown of NFATC3 partially suppresses the elevated ferroptosis sensitivity and lipid peroxidation of FAK OE or SRC OE B16-F10 cells. Cells as indicated were treated with erastin (30 μM) for 14 h for cell death measurement, or 12 h for lipid ROS measurement. Cell death and lipid ROS were measured as described in Fig. 1A. **T** Knockdown of SMAD4 partially suppresses the elevated ferroptosis sensitivity and lipid peroxidation of FAK OE or SRC OE B16-F10 cells. Cells as indicated were treated with erastin (30 μM) for 14 h for cell death measurement, or 12 h for lipid ROS measurement. Cell death and lipid ROS were measured as described in Fig. 1A. Data are derived from three independent experiments, and each value represents the mean ± SD. *P < 0.05, **P < 0.01, ***P < *0.001*, ****P < *0.0001*, *t* test. (*n* = 3).

Do JNK downstream transcriptional factors regulate the expression of ACSL4? We found that overexpression ATF2, NFATC1, NFATC3, or SMAD4 individually promoted the expression of ACSL4 on both protein level (Fig. 5A–D) and mRNA level (Fig. S5A–D). Knockdown of ATF2, NFATC1, NFATC3, or SMAD4 individually significantly suppressed ACSL4 expression on both protein level (Fig. 5E–H) and mRNA level (Fig. S5E–H). Furthermore, activation of SMAD4 by kartogenin (KGN) promoted the expression of ACSL4 on both protein level (Fig. S5I) and mRNA level (Fig. S5J) in both HT1080 and B16-F10 cells, and inhibition of NFATC1 by NDMC101 significantly suppressed ACSL4 expression on both protein level (Fig. S5K) and mRNA level (Fig. S5L) in HT1080 cells. We further analyzed the putative binding sites of these transcriptional factors at the promoter region of ACSL4 according to JASPAR transcription factor binding profile database [39]. And chromatin-IP assay confirmed that ATF2, NFATC1, NFATC3, and SMAD4 binds on the promote region of ACSL4 (Fig. 5I–L).

We further checked the requirement of ATF2, NFATC1, NFATC3, and SMAD4 on FAK/SRC-JNK axis regulated ferroptosis. We found that overexpression of ATF2, NFATC1, NFATC3, and SMAD4 individually in FAK KD cells, SRC KD cells or JNK1 KD cells restored their expression of ACSL4 (Fig. S5M–P) and their ferroptosis sensitivity (Fig. 5M–P). Knockdown of ATF2, NFATC1, NFATC3, and SMAD4 in FAK OE cells and SRC OE cells partially suppressed the elevated expression of ACSL4 (Fig. S5Q–T) and ferroptosis sensitivity in these cells (Fig. 5Q–T).

These data collectively suggest that the FAK/SRC-JNK axis promotes ferroptosis through ATF2, NFATC1, NFATC3, and SMAD4 mediated ACSL4 upregulation.

### JNK downstream transcriptional factors: c-Jun, ELK1, HSF1, and STAT3 suppress ferroptosis by inhibiting ACSL4 expression as a way of feedback regulation

**Fig. 6:**
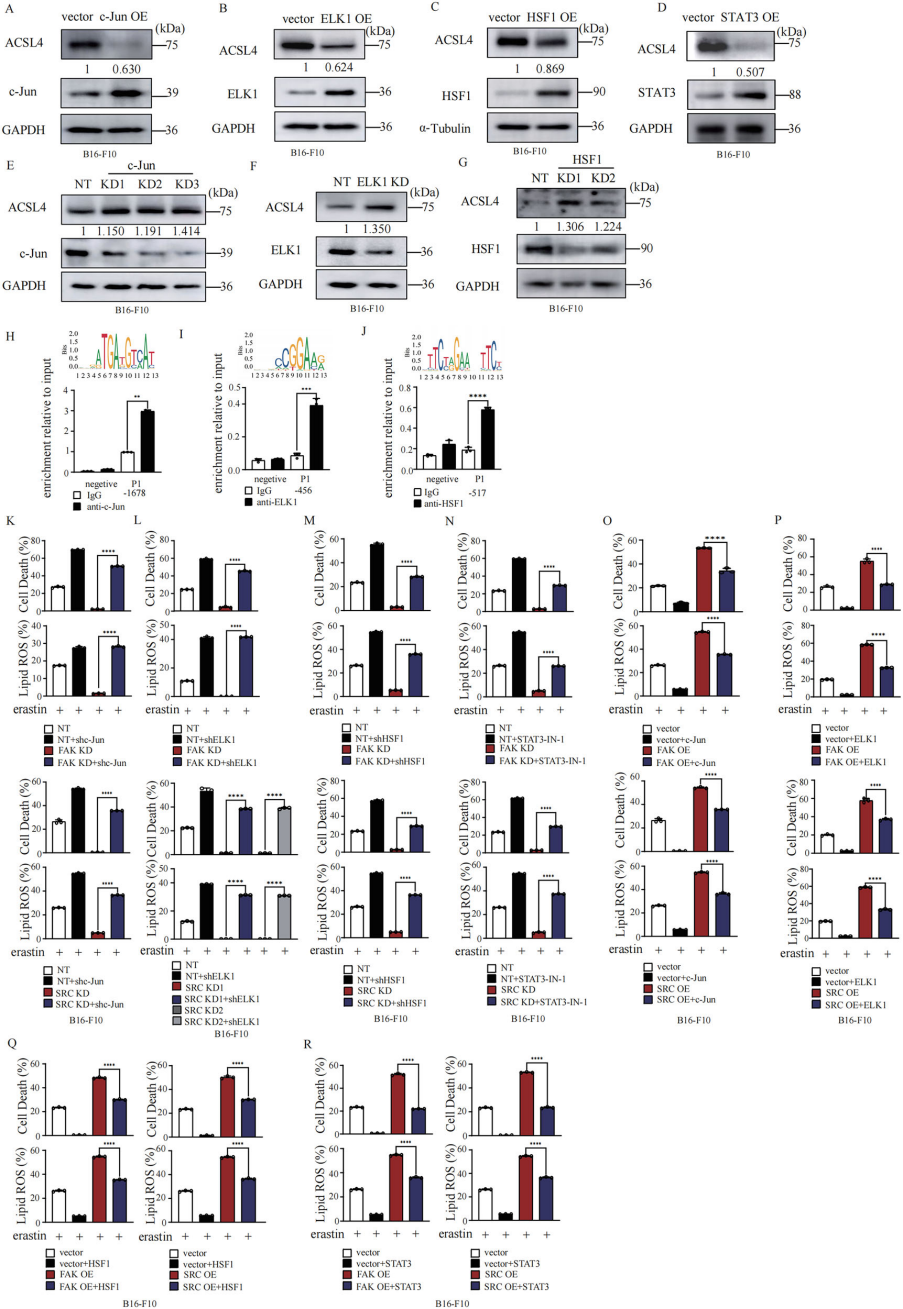
Four JNK downstream transcriptional factors: c-Jun, ELK1, and HSF1 suppress ferroptosis by inhibiting ACSL4 expression as a way of feedback regulation. **A** Overexpression of c-Jun suppresses the expression of ACSL4 in B16-F10 cells. Western blot images confirm the expression of the indicated proteins. **B** Overexpression of ELK1 suppresses the expression of ACSL4 in B16-F10 cells. Western blot images confirm the expression of the indicated proteins. **C** Overexpression of HSF1 suppresses the expression of ACSL4 in B16-F10 cells. Western blot images confirm the expression of the indicated proteins. **D** Overexpression of STAT3 suppresses the expression of ACSL4 in B16-F10 cells. Western blot images confirm the expression of the indicated proteins. **E** Knockdown of c-Jun promotes the expression of ACSL4 in B16-F10 cells. Western blot images confirm the expression of the indicated proteins. **F** Knockdown of ELK1 promoted the expression of ACSL4 in B16-F10 cells. Western blot images confirm the expression of the indicated proteins. **G** Knockdown of HSF1 promotes the expression of ACSL4 in B16-F10 cells. Western blot images confirm the expression of the indicated proteins. **H** c-Jun binds to the promoter region of ACSL4 in HT1080 cells. Chromatin-IP was performed using c-Jun antibody or control IgG. Values are percentage of input. Enrichment was normalized to the IgG control. The binding position on ACSL4 promoter and predicated binding sequence are shown as labelled. **I** ELK1 binds to the promoter region of ACSL4 in B16-F10 cells. Chromatin-IP was performed using ELK1 antibody or control IgG. Values are percentage of input. Enrichment was normalized to the IgG control. The binding position on ACSL4 promoter and predicated binding sequence are shown as labelled. **J** HSF1 binds to the promoter region of ACSL4 in HT1080 cells. Chromatin-IP was performed using HSF1 antibody or control IgG. Values are percentage of input. Enrichment was normalized to the IgG control. The binding position on ACSL4 promoter and predicated binding sequence are shown as labelled. **K** Knockdown of c-Jun rescues erastin induced ferroptosis sensitivity and lipid peroxidation of FAK KD or SRC KD B16-F10. B16-F10 cells as indicated were treated with erastin (30 μM) for 14 h for cell death measurement, or 12 h for lipid ROS measurement. Cell death and lipid ROS were measured as described in Fig. 1A. **L** Knockdown of ELK1 rescues erastin induced ferroptosis sensitivity and lipid peroxidation of FAK KD or SRC KD B16-F10. B16-F10 cells as indicated were treated with erastin (30 μM) for 14 h for cell death measurement, or 12 h for lipid ROS measurement. Cell death and lipid ROS were measured as described in Fig. 1A. **M** Knockdown of HSF1 rescues erastin induced ferroptosis sensitivity and lipid peroxidation of FAK KD or SRC KD B16-F10. B16-F10 cells as indicated were treated with erastin (30 μM) for 14 h for cell death measurement, or 12 h for lipid ROS measurement. Cell death and lipid ROS were measured as described in Fig. 1A. **N** Inhibition of STAT3 by STAT3-IN-1 (10 μM) rescues erastin induced ferroptosis sensitivity and lipid peroxidation of FAK KD or SRC KD B16-F10. B16-F10 cells as indicated were treated with erastin (30 μM) for 14 h for cell death measurement, or 12 h for lipid ROS measurement. Cell death and lipid ROS were measured as described in Fig. 1A. **O** Overexpression of c-Jun partially suppresses the elevated ferroptosis sensitivity and lipid peroxidation in FAK OE or SRC OE B16-F10 cells. B16-F10 cells as indicated were treated with erastin (30 μM) for 14 h for cell death measurement, or 12 h for lipid ROS measurement. Cell death and lipid ROS were measured as described in Fig. 1A. **P** Overexpression of ELK1 partially suppresses the elevated ferroptosis sensitivity and lipid peroxidation in FAK OE or SRC OE B16-F10 cells. B16-F10 cells as indicated were treated with erastin (30 μM) for 14 h for cell death measurement, or 12 h for lipid ROS measurement. Cell death and lipid ROS were measured as described in Fig. 1A. **Q** Overexpression of HSF1 partially suppressed the elevated ferroptosis sensitivity and lipid peroxidation in FAK OE or SRC OE B16-F10 cells. B16-F10 cells as indicated were treated with erastin (30 μM) for 14 h for cell death measurement, or 12 h for lipid ROS measurement. Cell death and lipid ROS were measured as described in Fig. 1A. **R** Overexpression of STAT3 partially suppresses the elevated ferroptosis sensitivity and lipid peroxidation in FAK OE or SRC OE B16-F10 cells. B16-F10 cells as indicated were treated with erastin (30 μM) for 14 h for cell death measurement, or 12 h for lipid ROS measurement. Cell death and lipid ROS were measured as described in Fig. 1A. All data are mean ± SD. from *n* = 3 biological replicates. *P < 0.05, **P < 0.01, ***P < *0.001* by two-tailed t-test.

Besides to these positive regulators of ferroptosis: ATF2, NFATC1, NFATC3, and SMAD4, we also determined how c-Jun, ELK1, HSF1 or STAT3 negatively regulate ferroptosis. We found that overexpression of c-Jun, ELK1, HSF1 or STAT3 individually suppressed expression of ACSL4 on both protein level (Fig. 6A–D) and mRNA level (Fig. S6A–D). Knockdown of c-Jun, ELK1 and HSF1 individually significantly increased ACSL4 expression (Fig. 6E–G) and mRNA level (Fig. S6E–G). Furthermore, activation of HSF1 by HSF1A suppressed the expression of ACSL4 on both protein level (Fig. S6H) and mRNA level (Fig. S6I). Inhibition of HSF1 by DTHIB significantly promoted ACSL4 expression on both protein level (Fig. S6J) and mRNA level (Fig. S6K). Inhibition of STAT3 by STAT3-IN-1 significantly promoted ACSL4 expression on both protein level (Fig. S6L) and mRNA level (Fig. S6M). We further analyzed the putative binding sites of these ferroptosis negative regulators at the promoter region of ACSL4 according to JASPAR transcription factor binding profile database. The chromatin-IP assay confirmed that c-Jun, ELK1 and HSF1 binds at the promote region of ACSL4 (Fig. 6H–J).

We further determined the role of these downstream transcriptional factors in FAK/SRC-JNK axis regulated ferroptosis. We found that knockdown of c-Jun, ELK1, HSF1 or inhibition of STAT3 individually in FAK KD cells or SRC KD cells restored their expression of ACSL4 (Fig. S6N–Q) and their ferroptosis sensitivity (Fig. 6K–N). Overexpression of c-Jun, ELK1, HSF1 or STAT3 individually in FAK OE cells and SRC OE cells blocked the elevated expression of ACSL4 (Fig. S6R–U) and ferroptosis sensitivity (Fig. 6O–R) in these cells.

These data collectively suggest that c-Jun, ELK1, HSF1 and STAT3 negatively regulate ferroptosis by blocking the expression of ACSL4.

Furthermore, we analyzed the binding kinetics of activators (ATF2, NFATC1, NFATC3, and SMAD4) vs. repressors (c-Jun, ELK1 and HSF1) with ChIP assay. Interestingly, we found that the pro-ACSL4 transcription factors bind more rapidly and robustly, while the repressors c-Jun and HSF1 bind slowly and erastin inhibits the binding of ELK1 to ACSL4 promoter upon erastin treatment, suggesting the repressors may act as a delayed negative feedback mechanism (Fig. S6V).

### Oncogenic FAK/SRC-JNK Axis promotes cancer cells ferroptosis in vivo

**Fig. 7:**
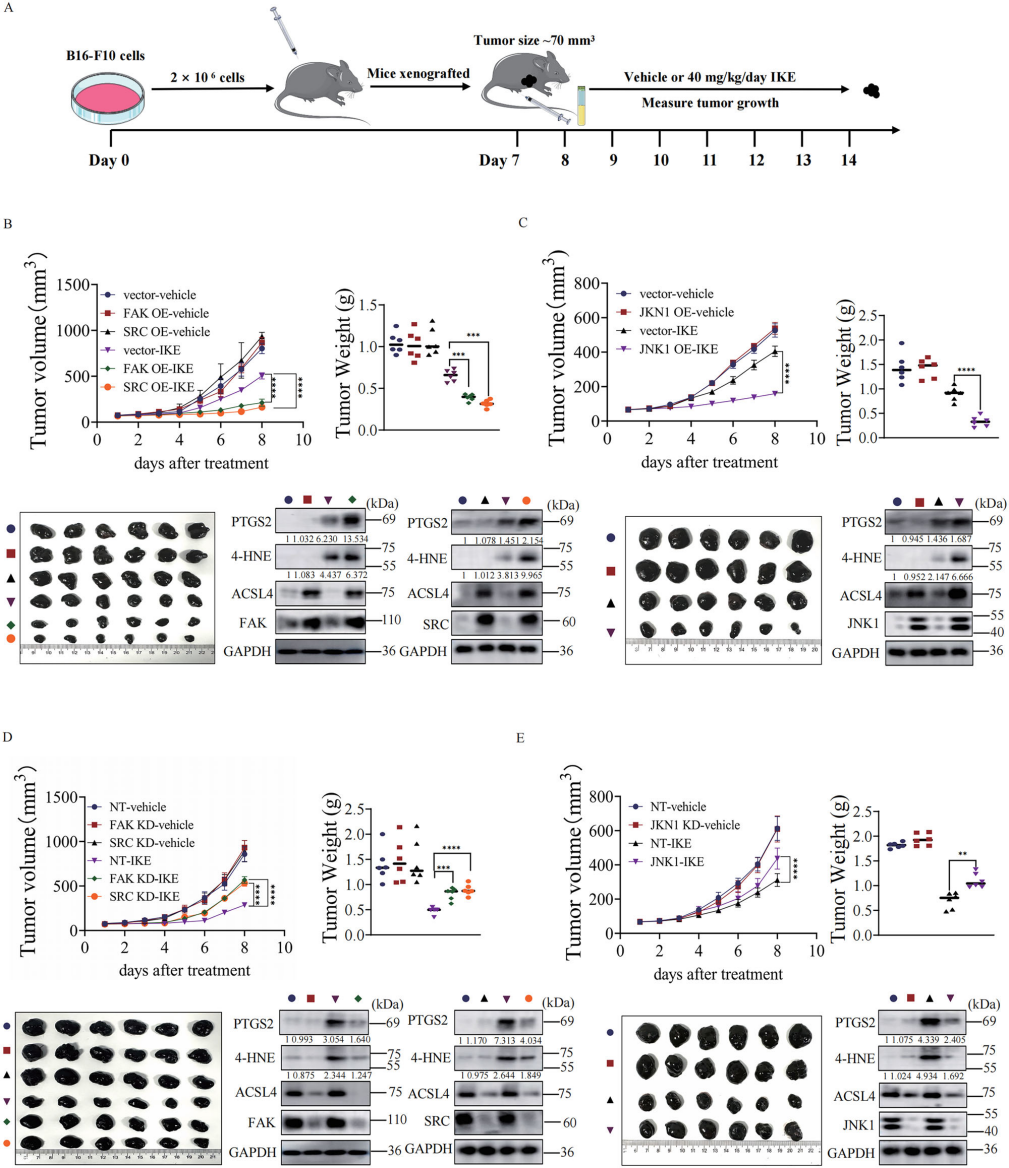
Oncogenic FAK/SRC-JNK Axis promotes cancer cells ferroptosis in vivo. **A** Schematic illustration of the experimental design of the mouse xenograft model. **B** Overexpression of FAK or SRC sensitizes cancer cells to ferroptosis-inducing tumor therapy in a mouse xenograft model. Mouse xenograft model experiments with indicated stable B16-F10 cells were performed as described in Fig.7A. Tumor volume was measured daily. Tumor volume post-IKE treatment is shown and tumors were photographed and weighted after 7 days IKE treatment. Western blot analysis of indicated proteins expression in tumor tissue. **C** Overexpression of JNK1 sensitizes cancer cells to ferroptosis-inducing tumor therapy in a mouse xenograft model. Mouse xenograft model experiments with indicated stable B16-F10 cells were performed as described in Fig. 7A and tumors were measured as described in Fig. 7B. Western blot analysis of indicated proteins expression in tumor tissue. **D** Knockdown of FAK or SRC confers resistance to ferroptosis-inducing tumor therapy in a mouse xenograft model. Mouse xenograft model experiments with indicated stable B16-F10 cells were performed as described in Fig. 7A and tumors were measured as described in Fig. 7B. Western blot analysis of indicated proteins expression in tumor tissue. **E** Knockdown of JNK1 confers resistance to ferroptosis-inducing tumor therapy in a mouse xenograft model. Mouse xenograft model experiments with indicated stable B16-F10 cells were performed as described in Fig. 7A and tumors were measured as described in Fig. 7B. Western blot analysis of indicated proteins expression in tumor tissue. All data are mean ± SD. *n* = 6 biological replicates. *P < 0.05, **P < 0.01, ***P < *0.001*, ****P < *0.0001* by two-tailed t-test.

FAK/SRC-JNK signaling plays crucial roles in cancer development. Tumor tissues typically have higher levels of FAK/SRC-JNK expression compared to normal adjacent tissue [40] (Fig. S7A). And high expression of FAK/SRC-JNK is corelated with poor overall survival in different type of cancer patients [40] (Fig. S7B). We thus explored whether FAK/SRC-JNK could be a good biomarker for ferroptosis-inducing cancer therapy. In mouse xenograft model (Fig. 7A), higher expression of FAK, SRC or JNK sensitizes melanoma to IKE treatment (Fig. 7B, C) and lower expression of FAK, SRC, or JNK decreases the sensitivity of melanoma to IKE treatment (Fig. 7D, E). Furthermore, we collected tumor tissues from our mouse xenograft model and measured key ferroptosis markers. Western blot analysis confirmed that tumors with higher FAK/SRC/JNK expression showed higher levels of ACSL4, 4-HNE and PTGS2 (COX2) after IKE treatment (Fig. 7B, C), while this induction was blunted in tumors with lower pathway expression (Fig. 7D, E). These data collectively suggest that FAK/SRC-JNK could be a biomarker for ferroptosis-inducing cancer therapy in certain cancer patients.

### Inhibition of FAK/SRC signaling Protects Pancreas from Acute Pancreatitis by Suppressing Ferroptosis

**Fig. 8:**
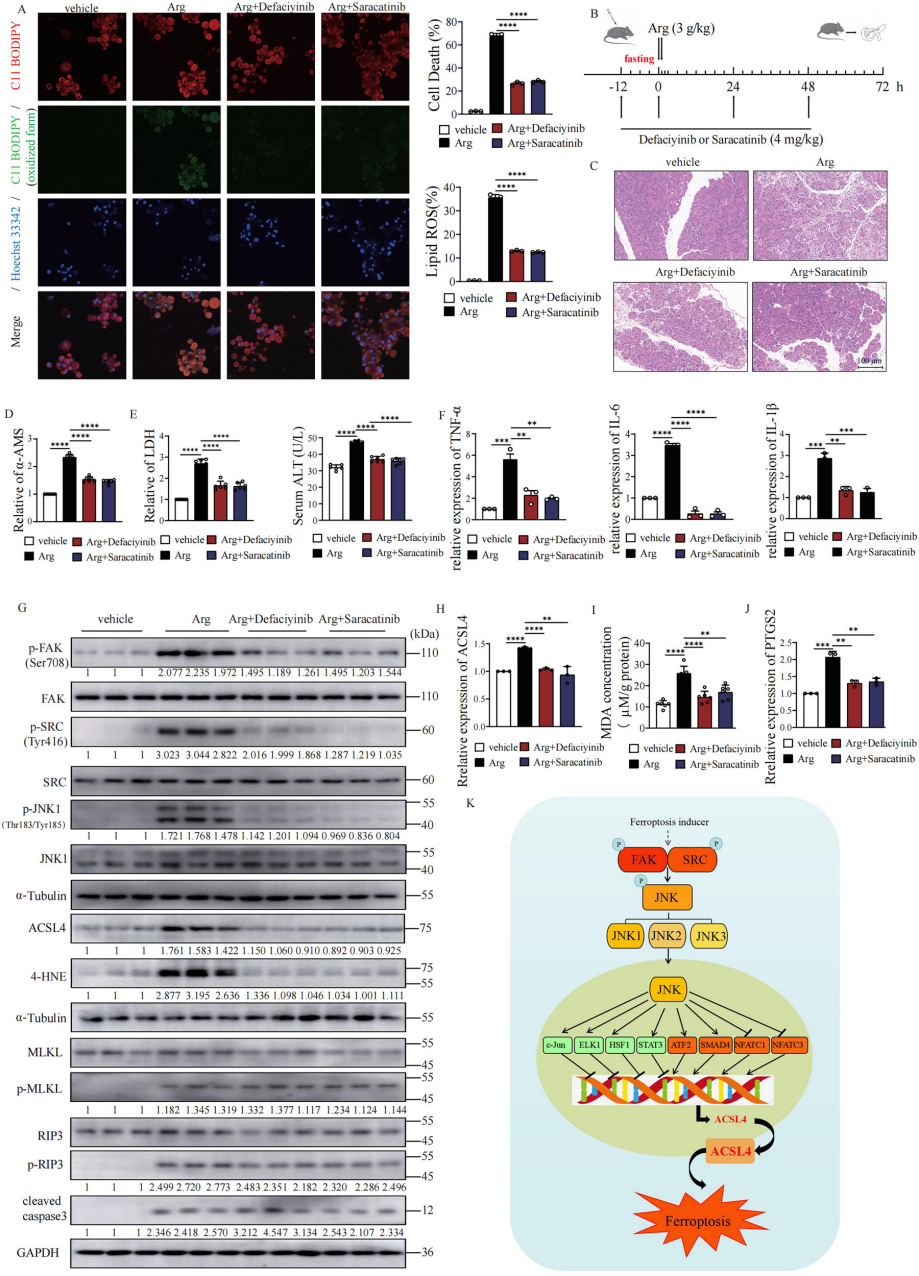
Inhibition of FAK/SRC signaling Protects Pancreas from Acute Pancreatitis by Suppressing Ferroptosis. **A** Defaciyinib or saracatinib treatment protects against arginine-induced cell death in isolated primary pancreatic acinar cells. **B** Schematic illustration of the experimental design of mouse pancreatitis model. **C** HE staining of pancreatic tissue. **D** Defaciyinib or saracatinib treatment alleviated the elevation of serum α-AMS in arginine induced acute pancreatitis. Mice were treated as Fig. 8A and serum α-AMS was measured with the α-AMS Assay Kit. **E** Defaciyinib or saracatinib treatment reduced the elevation of serum LDH and ALT in arginine induced acute pancreatitis. **F** Defaciyinib or saracatinib treatment suppressed the increase of pro-inflammatory cytokines (IL-1β, IL-6, and TNF-α) in arginine induced acute pancreatitis. RT-qPCR analyzes the expression of pro-inflammatory cytokines as indicated. **G** Defaciyinib or saracatinib treatment blocked the activation of FAK/SRC signal, lipid peroxidation and ACSL4 upregulation in arginine induced acute pancreatitis. Western blot analysis of indicated proteins expression in pancreatic tissue. **H** Defaciyinib or saracatinib treatment suppressed the increase of pancreatic tissue MDA in arginine induced acute pancreatitis. **I** Defaciyinib or saracatinib treatment blocked pancreatic tissue ACSL4 mRNA upregulation in arginine induced acute pancreatitis. **J** Defaciyinib or saracatinib treatment blocked pancreatic tissue PTGS2 mRNA upregulation in arginine induced acute pancreatitis. **K** The working model. All data are mean ± SD. *n* = 6 biological replicates. *P < 0.05, **P < 0.01, ***P < *0.001*, ****P < *0.001* by two-tailed t-test.

Acute pancreatitis (AP) is a severe inflammatory condition characterized by extensive tissue damage and cellular death. Emerging evidence implicates ferroptosis in the pathogenesis of AP [41]. Given our finding that the FAK/SRC signaling positively regulates ferroptosis, we investigated whether inhibition of this pathway could mitigate pancreatic injury in both arginine-induced cell death using isolated primary pancreatic acinar cells and a high-dose arginine-induced AP mouse model. We found that treatment with the FAK inhibitor Defactinib or the SRC inhibitor Saracatinib significantly protected primary acinar cells from arginine-induced cell death and lipid peroxidation (Fig. 8A). Arginine administration induced severe AP (Fig. 8B), as evidenced by HE staining (Fig. 8C), a marked increase of serum amylase (AMS, Fig. 8D), lactate dehydrogenase (LDH) and alanine aminotransferase (ATL) (Fig. 8E), alongside elevated expression of pro-inflammatory cytokines (TNF-α, IL-6 and IL-1β) in pancreatic tissue (Fig. 8F).

To further explore the therapeutic potential of targeting FAK/SRC signaling in AP, we evaluated the effects of FAK/SRC signaling inhibitors: defactinib and saracatinib. Both defactinib and saracatinib effectively alleviated arginine-induced AP, as demonstrated by HE staining, reduced serum level of AMS, LDH and ALT, and decreased expression of inflammatory cytokines (Fig. 8C–F). Arginine-induced AP was associated with elevated levels of 4-hydroxynonenal (4-HNE) and MDA, and upregulated expression of ACSL4 and PTGS2 (Fig. 8G–J), the biomarkers of lipid peroxidation and ferroptosis. More importantly, Arginine treatment induced significantly phosphorylation of FAK, SRC and JNK (Fig. 8G). Co-treatment with inhibitors of FAK/SRC signaling suppressed these changes (Fig. 8G–J), further supporting the involvement of ferroptosis and FAK/SRC signaling in AP pathogenesis. Interestingly, we also observed cleaved caspase-3 and phosphorylation of RIP3 and MLKL in pancreatic tissue from our mouse model, suggesting the involvement of apoptosis and necroptosis in acute pancreatitis. However, FAK inhibitor defactinib or SRC inhibitor saracatinib treatment has no significant effect on the level of cleaved caspase-3 and phosphorylation of RIP3 and MLKL, suggesting FAK/SRC signaling specifically regulating ferroptosis but not apoptosis or necroptosis in acute pancreatitis. Our findings highlight the critical role of ferroptosis in AP and suggest that pharmacological inhibition of FAK/SRC signaling represents a promising therapeutic strategy for mitigating pancreatic injury in AP.

## Discussion

In this study, we have uncovered a critical and novel role for the oncogenic FAK/SRC-JNK signaling axis as a central regulator of ferroptosis. We demonstrate that this pathway exerts its pro-ferroptotic effect primarily through the transcriptional upregulation of ACSL4, a key enzyme that primes cellular membranes for peroxidation. Crucially, we delineate a complex transcriptional network downstream of JNK, revealing that specific transcription factors (ATF2, NFATC1, NFATC3, SMAD4) act as positive regulators by directly enhancing ACSL4 expression, while another set (c-Jun, ELK1, HSF1, STAT3) function as negative regulators by repressing it. This dual regulatory mechanism unveils a previously unappreciated layer of complexity in ferroptotic signaling, suggesting a dynamic and finely-tuned balance that determines cellular fate in response to ferroptosis stress (Fig. 8K).

Our findings have profound implications for cancer biology and therapy. The FAK/SRC pathway is frequently hyperactivated in a wide range of advanced and metastatic cancers, where it promotes survival, proliferation, and therapy resistance. Paradoxically, we show that this very oncogenic activation renders cancer cells more susceptible to ferroptosis. This creates a compelling therapeutic opportunity: tumors reliant on FAK/SRC signaling may be uniquely vulnerable to ferroptosis-inducing agents (FINs). Our in vivo xenograft data robustly support this concept, showing that elevated FAK, SRC, or JNK levels sensitize tumors to IKE treatment, while their knockdown confers resistance. Therefore, the activation status of the FAK/SRC-JNK-ACSL4 axis could serve as a powerful predictive biomarker for patient stratification in clinical trials involving FINs, such as those targeting GPX4 or system xc⁻. Patients with tumors exhibiting high pathway activity might be prime candidates for such therapies.

Furthermore, the identification of specific antagonistic transcription factors provides a mechanistic explanation for the heterogeneous response to ferroptotic stimuli often observed between and within cancers. Two potential mechanisms could determine the net pro-ferroptotic or anti-ferroptotic outcome. Firstly, the cellular and tissue context, such as the relative abundance and activation kinetics of these TFs in different tissues or under different stresses, likely dictates the final transcriptional output for ACSL4 and ferroptosis sensitivity. The second potential mechanism is that the possibility of competition for binding sites on the ACSL4 promoter (e.g., the binding motifs of ATF2 and c-Jun are same) or cooperative binding between specific TFs (e.g., among the positive regulators) as a mechanism to favor a pro-ferroptotic signal. The balance between pro-ferroptotic ATF2, NFATC1, NFATC3 or SMAD4 and anti-ferroptotic c-Jun, ELK1, HSF1 or STAT3 in a given tumor could dictate its ultimate sensitivity. For example, the FAK inhibitor defactinib synergises with erastin when co-delivered in an injectable hydrogel for pancreatic ductal adenocarcinoma [42], this may be due to a high expression of downstream anti-ferroptotic transcription factors. This opens avenues for novel combination therapies. For instance, in a tumor where high FAK/SRC signaling is counteracted by high STAT3 activity, co-administration of a FIN with a STAT3 inhibitor could synergistically induce ferroptotic cell death. Similarly, pharmacological activation of HSF1, a known pro-survival factor in cancer, might be a mechanism of acquired resistance to ferroptosis, suggesting HSF1 inhibitors could be used to re-sensitize resistant tumors.

Beyond oncology, our work illuminates a promising therapeutic strategy for acute pancreatitis (AP), a condition with limited treatment options and significant morbidity. We convincingly demonstrate that arginine-induced AP is associated with activation of the FAK/SRC-JNK pathway, elevated lipid peroxidation, and ACSL4 upregulation. Most importantly, pharmacological inhibition of FAK or SRC with defactinib or saracatinib significantly ameliorated pancreatic injury, suppressed inflammation, and reduced ferroptotic markers. This positions ferroptosis not merely as a bystander but as a central executioner of cellular damage in AP and identifies FAK/SRC inhibitors as a novel class of potential therapeutic agents for this condition. It suggests that targeting ferroptosis could be beneficial in other inflammatory diseases where tissue damage is driven by similar mechanisms.

Several intriguing questions arise from our work. First, the precise mechanism by which ferroptosis inducers like erastin activate FAK and SRC remains to be elucidated. These phosphorylation events indicate FAK/SRC pathway activation upon ferroptotic stress, but whether this activation is relevant for amplifying the death signal should be further investigated. Is this a direct effect on integrin signaling or a consequence of broader membrane or oxidative stress? Second, the temporal and contextual regulation of the opposing transcription factors is unclear. What signals determine whether the pro- or anti-ferroptotic JNK transcriptome dominates in a given cellular context? Third, while we focused on ACSL4, it is possible that the FAK/SRC-JNK axis modulates other facets of ferroptosis, such as iron metabolism or GPX4 expression, which warrants further investigation.

A limitation of our study is its primary reliance on mouse models and cell lines. Future research should validate these findings in human patient samples—correlating FAK/SRC/JNK/ACSL4 pathway activity with clinical outcomes and response to therapy. Additionally, developing more specific inhibitors for the individual transcription factors identified (e.g., specific NFATC or SMAD4 activators) would allow for more precise therapeutic manipulation of this pathway.

In conclusion, we have defined the FAK/SRC-JNK-ACSL4 axis as a fundamental regulatory node controlling ferroptosis. This pathway functions as a double-edged sword: its oncogenic activation promotes cancer progression but simultaneously creates an Achilles’ heel of ferroptosis vulnerability. Conversely, its inhibition protects against inflammatory tissue damage in AP. By revealing the intricate transcriptional circuitry that governs this process, our work not only expands the fundamental understanding of ferroptotic regulation but also provides a robust rationale for targeting this pathway therapeutically in both cancer and inflammatory disease.

## Material and Methods

### Reagents and antibodies

Primary antibodies used were anti-FAK (proteintech, Cat#66258-1-Ig), anti-SRC (proteintech, Cat#60315-1-Ig), anti-JNK1 (proteintech, Cat#66210-1-Ig), anti-JNK2 (PTMALO, Cat#PTM-6948), anti-ACSL4 (SANTA CRUZ, Cat#sc365230), anti-SMAD4 (proteintech, Cat#10231-1-AP), anti-SMAD4 (MCE, Cat#HY-P80326), anti-Phospho-FAK-Ser708 (Wanleibio, Cat#WL02764), anti-Phospho-SRC-Tyr530 (Wanleibio, Cat#WL02114), anti-Phospho-JNK-Thr183/Tyr185 (Wanleibio, Cat#WL01813), anti-GAPDH (proteintech, Cat#60004-1-Ig). anti-NFATC1 (Wanleibio, Cat#WL01632), anti-NFATC1 (SANTA CRUZ, Cat#sc-7294), anti-NFATC3 (SANTA CRUZ, Cat#sc-8405), anti-ATF2 (SANTA CRUZ, Cat#sc-242), anti-ELK1 (proteintech, Cat#27420-1-AP), anti-ELK1 (PTM BIO, Cat#20042), anti-STAT3 (SANTA CRUZ, Cat#sc-8019), anti-p-ELK1 (SANTA CRUZ, Cat#sc-8406), anti-p-NFATC3 (SANTA CRUZ, Cat#sc-365785), anti-c-Jun (CST, Cat#9165), anti-c-Jun (proteintech, Cat#24909-1-AP), anti-p-c-Jun (SANTA CRUZ, Cat#sc-8322), anti-p-ATF2 (SANTA CRUZ, Cat#sc-8398), anti-HSF1 (SANTA CRUZ, Cat#sc-17757), anti-SLC7A11 (proteintech, Cat#26864-1-AP), anti-GPX4 (abcam, Cat#ab231174), anti-alpha Tubulin (proteintech, Cat#11224-1-AP), anti-ATP1A1 (proteintech, Cat#14418-1-AP), anti-Histone H3 (Wanleibio, Cat#WL0984a), anti-MBOAT1 (proteintech, Cat#25615-1-AP), anti-MBOAT2 (abcepta, Cat#AP17786c), anti-DHODH (proteintech, Cat#14877-1-AP), anti-ALOX15 (abcam Cat#244205), anti-4-Hydroxynonenal (Thermo fisher, Cat# MA5-27570), anti-NCOA4 (proteintech, Cat#10727-1-AP), anti-FTH1 (HUABIO, Cat#ET1705-55) and anti-FTL1 (Affinity, Cat#DF6604), anti-RIP3 (HUABIO cat#HA722183), anti-p-RIP3 (HUABIO cat#HA721428), anti-caspase3 (proteintech, Cat#19677-1-AP), anti-p-MLKL (BOSTER cat#P00535), anti-MLKL (proteintech, Cat#66675-1-Ig)

Chemical reagents used were erastin (Selleck, Cat#S7242), Defactinib (TargetMOL, Cat#1073154-85-4), Saracatinib (Selleck, Cat#AZDO530), Imidazole ketone erastin (Selleck, Cat#S8877), Kartogenin (Adoop Bioscience, Cat#A12926), DTHIB (TargetMOL, Cat#897326-30-6), HSF1A (TargetMOL, Cat#1196723-93-9), STAT3-IN-1 (TargetMOL, Cat#2059952-75-7), NDMC101 (TargetMOL, Cat#1308631-40-4), BODIPY C11 (Thermo Fisher, Cat# D3861). Hematoxylin-Eosin (H&E) HD Constant Dye Kit (Servicebio, Cat# G1076), PI (Sigma-Aldrich, Cat#25535-16-4), Protein A/G AgaroseThermo Fisher, Cat#20421, Protein A/G Magnetic Beads (MCE,Cat#HY-K0202), TRIzol (Thermo Fisher, Cat# 15596026) Xylene (SCRC, Cat# 10023418), Normal Butanol (SCRC, Cat# 100052190), Environmental Friendly Dewaxing Transparent Liquid (Servicebio, Cat# G1128-1L), Paraformaldehyde Fixative (Neutral) (Servicebio, Cat# G1101), Neutral Gum (SCRC, Cat# 10004160), L-Arginine (Sigma, Cat#74-79-3).

### Cell culture

HT1080, B16-F10 and HEK293T were purchased from ATCC. All mammalian cells are maintained in DMEM with high glucose, sodium pyruvate (1 mM), glutamine (4 mM), penicillin (100 U/mL), streptomycin (0.1 mg/mL) and 10% (v/v) FBS at 37 °C and 5% CO_2_. Cell death was analyzed by PI (100 ng/mL) staining coupled with microscopy or flow cytometry.

### Bioactive Compound Library Screening

The Bioactive Compound Library (APExBio, Cat# L1022P) consist with 4417 bioactive compounds. Stock solutions (10 mM) were prepared in DMSO and arrayed in 96-well plates. HT1080 cells seeded in 24-well plates with a density of 8 × 10^4^ per well were treated with erastin (10 μM) plus individual compound (10 μM) from the library for 18 h. Cell death was measured by PI staining followed by microscopy or flow cytometry.

### Measurement of lipid peroxidation

Lipid peroxidation was analyzed by flow cytometry according to previous study. Briefly, cells were seeded at a density of 1.2 × 10^5^ per well in a 12-well dish and cultured overnight in DMEM. Cells were treated as indicated, 5 μM BODIPY C11 was added into cell culture medium and incubated for 30 min. Excess BODIPY C11 was then removed by washing the cells with PBS twice. BODIPY C11 labelled cells were resuspended in PBS plus 2% FBS. Oxidation of BODIPY C11 resulted in a move of the fluorescence emission peak from 590 nm to 510 nm proportional to lipid ROS generation and was analyzed using a flow cytometer.

### Plasmids

Plasmids for overexpression of FAK, SRC, JNK1, JNK2, ACSL4, ATF2, NFATC1, NFATC3, SMAD4, c-Jun, ELK1, HSF1, and STAT3 are purchased from Miaoling plasmid platform. Mission lentiviral shRNA clones targeting human FAK, human c-SRC, mouse FAK, mouse c-SRC, mouse JNK1, mouse JNK2, human ELK1, mouse ELK1, mouse c-Jun, human SMAD4, mouse SMAD4, mouse NFATC1, mouse ATF2, mouse HSF1 and non-targeting control construct were purchased from sigma-aldrich. The TRC ID for the shRNA targeting genes are as following:

human FAK-shRNA-1: TRCN0000121129; human FAK-shRNA-2: TRCN0000121207;

human FAK-shRNA-3: TRCN0000121318, human SRC-shRNA-1: TRCN0000038150;

human SRC-shRNA-2: TRCN0000195339; human SRC-shRNA-3: TRCN0000038149,

mouse FAK-shRNA-1: TRCN0000023485; mouse SRC-shRNA-1: TRCN0000278660,

mouse SRC-shRNA-2: TRCN0000023594; mouse JNK1-shRNA-1: TRCN0000301243;

mouse JNK1-shRNA-2: TRCN0000310849; mouse JNK1-shRNA-3: TRCN0000304297;

mouse JNK2-shRNA-1: TRCN0000012592; mouse JNK2-shRNA-2: TRCN0000348049;

mouse NFATC1-shRNA-1: TRCN0000081925; mouse NFATC1-shRNA-2: TRCN0000081923;

mouse NFATC1-shRNA-3: TRCN0000081924; mouse NFATC3-shRNA-1: TRCN0000097129;

mouse NFATC3-shRNA-2: TRCN0000097130; mouse NFATC3-shRNA-3: TRCN0000097132;

mouse ATF2-shRNA-1: TRCN0000082081; mouse ATF2-shRNA-2: TRCN0000365933;

mouse HSF1-shRNA-1: TRCN0000008502; mouse HSF1-shRNA-2: TRCN0000008504;

### Separation of nuclear and cytoplasmic protein

To get nuclear and cytoplasmic protein fraction, 2 × 10^7^ cells were collected and resuspended in 500 μL of separation buffer (20 mM HEPES, 10 mM KCl, 2 mM MgCl_2_, 1 mM EDTA, 1 mM EGTA, 1 mM DTT, 1% Proteases Inhibitor Cocktail). Incubate on ice for 15 min. The cells were homogenized suspension 10 times using a 1 mL syringe through a No. 27 needle. Centrifuge the sample at a speed of 3000 rpm for 5 min. The precipitate after centrifugation will contain the cell nucleus, and the supernatant will contain the cytoplasm, cell membrane and mitochondria. Wash the remaining nuclear precipitate with 500 μL of separation buffer. Disperse the sediment with a pipette and then homogenize it 10 times through a No. 25 gauge needle. Centrifuge at a speed of 3000 rpm for another 10 min. Discard the supernatant and retain the precipitate containing the cell nucleus. Resuspend the precipitate in TBS with 0.1% SDS. The suspension was subjected to a brief ultrasonic treatment to shear genomic DNA and homogenize the lysate.

### Separation of plasma membrane

To get plasma membrane fraction, 2 × 10^7^ cells were collected and lysed in cold assay buffer A (1 mM KCl, 5 mM NaCl, 3 mM MgCl_2_, 50 mM Hepes, 1 mM DTT, 1% Proteases Inhibitor Cocktail). Cells were frozen and thawed repeatedly in liquid nitrogen for 5 times, then centrifuged at 5000 rpm for 10 min at 4 °C and the supernatant was centrifuged at 12,000 rpm for 10 min at 4 °C. The pellet was resuspended in cold assay buffer B (1 mM KCl, 5 mM NaCl, 3 mM MgCl_2_, 50 mM Hepes, 1 mM DTT, 1 mM EGTA, 1% Proteases Inhibitor Cocktail) and centrifuged at 12,000 rpm for 10 min at 4 °C. Resuspend pellets in cold assay buffer C (50 mM Tris-HCl, pH 7.0, 1% Protease Inhibitor Cocktail) to get plasma membrane fraction.

### Chromatin immunoprecipitation (ChIP) assay

The ChIP experiment was performed as previous study. Briefly, 4 × 10^7^ HT1080 cells or B16 cells were crosslinked in 0.75% formaldehyde for 10 min. 125 mM glycine was added and incubated for 5 min. Cells were washed with cold PBS once and then harvested in PBS and sonicated with an ultrasonic homogenizer on ice for 10 min at 30% power (6 pulses, 30 s on and 30 s off) to shear DNA into fragments with an average size of 200–1000 bp. 50 µL sonicated sample was used to measure the DNA concentration and fragment size. 100 uL cell lysates were incubated overnight with 100 µL protein A/G agarose (Thermo, Cat#20421) banded with specific antibodies or IgG antibody (proteintech, Cat#B900620). Beads were collected, washed and treated with proteinase K for 4 h at 65 °C and RNase for 1 h at 37 °C. DNA was purified with a PCR purification kit (Thermo, Cat#K0702) and eluted with 20 µL ddH2O. DNA fragments were assessed by quantitative PCR by using universal SYBR qPCR Master Mix Kit (Vazyme, Cat#Q511-03) using the primer sequences listed in Supplementary material 1. Samples were normalized to input DNA.

### RT–qPCR

Total RNA was extracted using TRIzol reagent (Thermo Fisher, Cat# 15596026) according to the manufacturer’s instructions. cDNA was synthesized using the cDNA Synthesis Kit (Takara, Cat# RR047A) according to the manufacturer’s instructions. RT–qPCR was performed with ChamQ Universal SYBR qPCR Master Mix (Vazyme, Cat# Q711-02) in a Real-Time PCR system (Applied Biosystems). Primer sequences are listed in the Supplementary table.

### Mouse xenograft model

1 × 10^6^ B16 mouse melanoma cells were suspended in 100 μL PBS and injected subcutaneously into 6-week-old male C57BL/6 mouse purchased from Liaoning Changsheng biotechnology Co., Ltd. Tumor was measured every day and volume was calculated as length×(width) [2]. When the tumor volume reached 25 mm^3^, 4 mg/kg imidazole ketone erastin was administered once a day last a week. Tumor weight was measured on the 8th day after mice were sacrificed.

### Isolation and Primary Culture of Pancreatic Acinar Cells

Following euthanasia, the pancreas was aseptically harvested from C57 mice and immediately placed in a sterile culture dish containing ice-cold wash medium (PBS without Ca²⁺/Mg²⁺). The tissue was rinsed several times with the pre-chilled wash medium to remove blood and contaminants. The pancreas was meticulously minced into small fragments of approximately 1–2 mm³. The tissue fragments were transferred to a conical tube and centrifuged at 450 rpm for 2 min. The supernatant and any visible debris were carefully discarded. The pellet was resuspended in 3 mL of digestion solution containing 200 U/mL Type II collagenase. The mixture was pipetted gently to ensure even distribution of the tissue. Digestion was performed in a 37 °C incubator for a total of 25 min, with intermittent agitation. Specifically, the tube was incubated for 5-min intervals, repeated five times. Digestion was terminated immediately upon observation of medium turbidity. The resulting cell suspension was sequentially filtered through 100 and 40 μm cell strainers to remove undigested tissue fragments and large cell aggregates. The filtrate was collected and centrifuged at 450 rpm for 2 min to pellet the cells. The supernatant was discarded, and the cell pellet was washed twice with a termination solution (base medium supplemented with 5% FBS) to inactivate the collagenase. Finally, the purified acinar cells were resuspended in complete culture medium, consisting of base medium supplemented with 2.5% FBS, 1% Penicillin-Streptomycin, and 2.5 U/mL Soybean Trypsin Inhibitor. The cells were then ready for subsequent seeding and culture.

### Arginine-induced in vitro model in primary acinar cells

Primary acinar cells were treated with 75 mM L-arginine for 45 min. Following treatment, the medium was aspirated, and the cells were washed with phosphate-buffered saline (PBS) and subsequently cultured in fresh normal medium. Lipid ROS levels were assessed after 12 h of culture, and cell death was determined by PI staining at the 24-hour time point.

### Mice acute pancreatitis model

All animal studies were approved by the Animal Experimental Ethics Committee of Harbin Institute of Technology. The C57BL/6 mice were purchased from Liaoning Changsheng biotechnology Co., Ltd. Inducing L-Arginine acute pancreatitis in mice was performed according to previous study with some modification. Briefly, 8-week-old C57 mice were pretreated with (4 mg/kg) defactinib or saracatinib for 24 h and fasted for 12 h. Administer L-arginine (3 g/kg) solution with defactinib or saracatinib intra-peritoneally using syringe with 30 G 1/2” needle followed by second L-arginine solution alone i.p. injection after 1 h. (4 mg/kg) defactinib or saracatinib was i.p. injected twice at 24 and 48 h. Mice are kept under observation, allowed free access to feed and water, and sacrificed at 72 h. Blood is collected from heart using heparin treated syringes and is centrifuged at 4 °C. Plasma is stored at −80 °C for amylase, LDH, ALT and MDA analysis. Mice pancreas was harvested for mRNA and protein expression analysis.

### HE staining

The paraffin sections were immersed in sequence in Environmental Friendly Dewaxing Transparent Liquid I and II for dewaxing and hydration. The frozen sections were removed from the −20 °C refrigerator and restored to room temperature, fixed with tissue fixating solution for 15 min, and then rinsed with running water. The sections were then treated with HD constant staining pretreatment solution for 1 min. Put sections into Hematoxylin solution for 3–5 min. Then treat the section with Hematoxylin Differentiation solution, Hematoxylin Bluing solution. Place the sections in 95% ethanol for 1 min, Eosin dye for 15 s. Finally, dehydration and sealing.

### Malondialdehyde assay

High intracellular malondialdehyde (MDA) levels indicate lipid peroxidation. The intracellular MDA concentration in pancreas tissue was assessed using the MDA Assay Kit (Beyotime, Cat# S0131S) according to the manufacturer’s instructions.

### Lactate Dehydrogenase assay

Transfer of a 50 μL diluted sample from the Sample Plate to the Assay Plate, addition of 25 μL Assay Buffer A (4 mM INT (4×) in 0.2 M Tris-HCl) and 25 μL Assay Buffer B (6.4 mM NAD (4×) and 320 mM lithium lactate (4×) in 0.2 M Tris-HCl) and 0.0005 μL MPMS (150 mM MPMS in Tris buffer), followed by a 1 h incubation then addition of 50 μL Stop Solution (1 M Acetic acid) and measurement of absorbance at λ = 490 nm.

### Alanine Aminotransferase assay

The serum ALT concentration was assessed using the ALT Assay Kit (Jiancheng Bio, Cat# C009-3) according to the manufacturer’s instructions.

### Amylase assay

The serum α-AMS concentration was assessed using the α-AMS Assay Kit (Jiancheng Bio, Cat# C016-1-1) according to the manufacturer’s instructions.

### Statistical analysis

All statistical analyses were performed using Prism 8.5c GraphPad Software. *P* values were calculated with unpaired Student’s *t*-test. Data are presented as mean ± SEM from 3 independent experiments. *P* < 0.05 was set as the threshold for significance (*P < 0.05, **P < 0.01, ***P < *0.001*, ****P < *0.0001*).

## Supplementary information

Supplementary figure legend

Supplementary figure 1

Supplementary figure 2

Supplementary figure 3

Supplementary figure 4

Supplementary figure 5

Supplementary figure 6

Supplementary figure 7

uncropped westen blot image

## Data Availability

The data that support the findings of this study are available from the corresponding author (M Gao) upon reasonable request.
