# Supplementary material for: FAK/SRC-JNK axis promotes ferroptosis via upregulating ACSL4 expression
Source: Cell Death Dis. 2026 Mar 20;17(1):328. doi: 10.1038/s41419-026-08570-y (PMC13039192; doi:10.1038/s41419-026-08570-y)
Supplement: Supplementary file 1 — Supplementary figure legend [file 41419_2026_8570_MOESM1_ESM.docx]

**Figure S1. FAK/SRC complex positively regulate ferroptosis**

(A) Overview of the ferroptosis inhibitor screening in MEF cells. Cells cultured overnight in 24-well plates were treated with erastin (10 μM) plus individual small compound from the library for 12 h. Cells were stained with Propidium Iodide (PI) and cell death was analyzed by florescence microscopy. (B) Reconstitution of FAK back to FAK KD cells restores the sensitivity to ferroptosis and lipid peroxidation. Cells as indicated were treated with erastin (10 μM for HT1080 cells and 30 μM for B16-F10 cells) for 12 h for cell death measurement, or 10 h for lipid ROS measurement. Cell death and lipid ROS were measured as described in Figure 1A. Western blot images confirm the expression of the indicated protein. (C) Reconstitution of SRC back to SRC KD cells restores the sensitivity to ferroptosis and lipid peroxidation. Cells as indicated were treated with erastin (10 μM for HT1080 cells and 30 μM for B16-F10 cells) for 12 h for cell death measurement, or 10 h for lipid ROS measurement. Cell death and lipid ROS were measured as described in Figure 1A. Western blot images confirm the expression of the indicated protein. (D) Knockdown of FAK suppresses RSL3 induced cell death and lipid peroxidation. B16-F10 Cells as indicated were treated with 0.1 μM RSL3 for 14 h for cell death measurement, or 10 h for lipid ROS measurement. then cell death and lipid ROS were measured as described in Figure 1A. (E) Knockdown of SRC suppresses RSL3 induced cell death and lipid peroxidation. B16-F10 Cells as indicated were treated with 0.1 μM RSL3 for 14 h for cell death measurement, or 10 h for lipid ROS measurement. then cell death and lipid ROS were measured as described in Figure 1A.

All data are mean ± SD. from n = 3 biological replicates. **P < 0.05, **P < 0.01, ***P < 0.001, ***P < 0.0001* by two-tailed t-test.

**Figure S2. FAK/SRC promotes ferroptosis by upregulation of ACSL4**

1. Knockdown of FAK suppresses the mRNA level of ACSL4 in HT1080 cells and B16-F10 cells. RT-qPCR analysis of ACSL4 expression is shown. (B) FAK inhibitor defactinib (20 μM for 12 h) treatment suppresses the mRNA level of ACSL4 in HT1080 cells and B16-F10 cells. RT-qPCR analysis of ACSL4 expression is shown. (C) Reconstitution of FAK back to FAK KD cells restored the expression of ACSL4 in HT1080 cells and B16-F10 cells. Western blot images confirm the expression of the indicated protein. (D) Overexpression of FAK promotes the mRNA level of ACSL4 in HT1080 cells and B16-F10 cells. RT-qPCR analysis of ACSL4 expression is shown. (E) Knockdown of SRC suppresses the mRNA level of ACSL4 in HT1080 cells and B16-F10 cells. RT-qPCR analysis of ACSL4 expression is shown. (F) SRC inhibitor saracatinib (40 μM for 12 h) treatment suppresses the mRNA level of ACSL4 in HT1080 cells and B16-F10 cells. RT-qPCR analysis of ACSL4 expression is shown. (G) Reconstitution of SRC back to SRC KD cells restored the expression of ACSL4 in HT1080 cells and B16-F10 cells. Western blot images confirm the expression of the indicated protein. (H) Overexpression of SRC promotes the mRNA level of ACSL4 in HT1080 cells and B16-F10 cells. RT-qPCR analysis of ACSL4 expression is shown.

All data are mean ± SD. from n = 3 biological replicates. **P < 0.05, **P < 0.01, ***P < 0.001, ****P < 0.0001* by two-tailed t-test.

**Figure S3. JNK1/2 positively regulates ferroptosis via upregulation of ACSL4**

(A) Knockdown of JNK1 or JNK2 suppresses the mRNA level of ACSL4 in B16-F10 cells. RT-qPCR analysis of ACSL4 expression is shown. (B) Reconstitution of JNK1 back to JNK1 KD or reconstitution of JNK2 back to JNK2 KD cells rescue the sensitivity to ferroptosis and lipid peroxidation. Cells as indicated were treated with erastin (30 μM for B16-F10 cells) for 12 h for cell death measurement, or 10 h for lipid ROS measurement, then cell death and lipid ROS were measured as described in Figure 1A. Western blot images confirm the expression of the indicated protein. (C) Reconstitution of JNK1 back to JNK1 KD or reconstitution of JNK2 back to JNK2 KD cells restores the expression of ACSL4 in B16-F10 cells. Western blot images confirm the expression of the indicated protein. (D) Overexpression of JNK1 or JNK2 promotes the mRNA level of ACSL4 in B16-F10 cells. RT-qPCR analysis of ACSL4 expression is shown. (E) Overexpression of JNK1 does not change the expression of indicated ferroptosis regulators. Western blot images confirm the expression of the indicated protein. (F) JNK1 and JNK2 redundantly promote ACSL4 expression and enhance ferroptosis sensitivity. Indicated B16-F10 cells were treated with erastin as indicated for cell death measurement, or lipid ROS measurement as described in Figure 1A. Western blot images confirm the expression of the indicated proteins. (G) Knockdown of JNK1 suppresses RSL3 induced cell death and lipid peroxidation. B16-F10 Cells as indicated were treated with 0.1 μM RSL3 for 14 h for cell death measurement, or 10 h for lipid ROS measurement. then cell death and lipid ROS were measured as described in Figure 1A.

All data are mean ± SD. from n = 3 biological replicates. **P < 0.05, **P < 0.01, ***P < 0.001, ****P < 0.0001* by two-tailed t-test.

**Figure S4. JNK downstream transcriptional factors regulate cancer cells susceptibility to ferroptosis**

(A) SMAD4 activator kartogenin treatment promotes ferroptosis and lipid peroxidation. Cells as indicated were treated with erastin (10 μM for HT1080, 30 μM for B16-F10) with kartogenin (10 μM) or not for 12 h for cell death measurement , or 10 h for lipid ROS measurement. Cell death and lipid ROS were measured as described in Figure 1A. (B) NFATC3 inhibitor NDMC101 treatment suppresses ferroptosis and lipid peroxidation. Cells as indicated were treated with erastin (10 μM for HT1080, 30 μM for B16-F10) with NDMC101 (10 μM) or not for 14 h for cell death measurement , or 12 h for lipid ROS measurement. Cell death and lipid ROS were measured as described in Figure 1A. (C) STAT3 inhibitor STAT3-IN-1 treatment promotes ferroptosis and lipid peroxidation. Cells as indicated were treated with erastin (10 μM for HT1080, 30 μM for B16-F10) with STAT3-IN-1 (10 μM) or not for 12 h for cell death measurement , or 10 h for lipid ROS measurement. Cell death and lipid ROS were measured as described in Figure 1A. (D) HSF1 inhibitor DTHIB treatment promotes ferroptosis and lipid peroxidation. Cells as indicated were treated with erastin (10 μM for HT1080, 30 μM for B16-F10) with DTHIB (10 μM) or not for 12 h for cell death measurement , or 10 h for lipid ROS measurement. Cell death and lipid ROS were measured as described in Figure 1A. (E) HSF1 activator HSF1A treatment suppresses ferroptosis and lipid peroxidation. Cells as indicated were treated with erastin (10 μM for HT1080) with HSF1A (10 μM) or not for 14 h for cell death measurement , or 12 h for lipid ROS measurement. Cell death and lipid ROS were measured as described in Figure 1A.

All data are mean ± SD. from n = 3 biological replicates. **P < 0.05, **P < 0.01, ***P < 0.001, ****P < 0.0001* by two-tailed t-test.

**Figure S5. The FAK/SRC-JNK axis promotes ferroptosis via ATF2, SMAD4, NFATC1, and NFATC3, mediated ACSL4 upregulation**

(A) Overexpression of ATF2 promotes the mRNA level of ACSL4 in B16-F10 cells. RT-qPCR analysis of ACSL4 expression is shown. (B) Overexpression of NFATC1 promotes the mRNA level of ACSL4 in B16-F10 cells. RT-qPCR analysis of ACSL4 expression is shown. (C) Overexpression of NFATC3 promotes the mRNA level of ACSL4 in B16-F10 cells. RT-qPCR analysis of ACSL4 expression is shown. (D) Overexpression of SMAD4 promotes the mRNA level of ACSL4 in B16-F10 cells. RT-qPCR analysis of ACSL4 expression is shown. (E) Knockdown of ATF2 suppresses the mRNA level of ACSL4 in B16-F10 cells. RT-qPCR analysis of ACSL4 expression is shown. (F) Knockdown of NFATC1 suppresses the mRNA level of ACSL4 in B16-F10 cells. RT-qPCR analysis of ACSL4 expression is shown. (G) Knockdown of NFATC3 suppresses the mRNA level of ACSL4 in B16-F10 cells. RT-qPCR analysis of ACSL4 expression is shown. (H) Knockdown of SMAD4 suppresses the mRNA level of ACSL4 in B16-F10 cells. RT-qPCR analysis of ACSL4 expression is shown. (I) SMAD4 activator kartogenin (10 μM for 12h) treatment promotes ACSL4 expression in HT1080 and B16-F10 cells. Western blot images confirm the expression of the indicated protein. (J) SMAD4 activator kartogenin (10 μM for 12h) treatment promotes the mRNA level of ACSL4 in HT1080 and B16-F10 cells. RT-qPCR analysis of ACSL4 expression is shown. (K) NFATC3 inhibitor NDMC101 (10 μM for 12h) treatment suppresses ACSL4 expression in HT1080 cells. Western blot images confirm the expression of the indicated protein. (L) NFATC3 inhibitor NDMC101 (10 μM for 12h) treatment suppresses the mRNA level of ACSL4 in HT1080 cells. RT-qPCR analysis of ACSL4 expression is shown. (M) Overexpression of ATF2 in FAK KD, SRC KD or JNK1 KD B16-F10 cells rescues the expression of ACSL4. Western blot images confirm the expression of the indicated protein. (N) Overexpression of NFATC1 in FAK KD, SRC KD or JNK1 KD B16-F10 cells rescues the expression of ACSL4. Western blot images confirm the expression of the indicated protein. (O) Overexpression of NFATC3 in FAK KD, SRC KD or JNK1 KD B16-F10 cells rescues the expression of ACSL4. Western blot images confirm the expression of the indicated protein. (P) Overexpression of SMAD4 in FAK KD, SRC KD or JNK1 KD B16-F10 cells rescues the expression of ACSL4. Western blot images confirm the expression of the indicated protein. (Q) Knockdown of ATF2 partially suppresses the elevated expression of ACSL4 in FAK OE or SRC OE B16-F10 cells. Western blot images confirm the expression of the indicated protein. (R) Knockdown of NFATC1 partially suppresses the elevated expression of ACSL4 in FAK OE or SRC OE B16-F10 cells. Western blot images confirm the expression of the indicated protein. (S) Knockdown of NFATC3 partially suppresses the elevated expression of ACSL4 in FAK OE or SRC OE B16-F10 cells. Western blot images confirm the expression of the indicated protein. (T) Knockdown of SMAD4 partially suppresses the elevated expression of ACSL4 in FAK OE or SRC OE HT1080 cells. Western blot images confirm the expression of the indicated protein.

All data are mean ± SD. from n = 3 biological replicates. **P < 0.05, **P < 0.01, ***P < 0.001, ****P < 0.0001* by two-tailed t-test.

**Figure S6. JNK downstream transcriptional factors: c-Jun, ELK1, and HSF1 suppress ferroptosis by inhibiting ACSL4 expression as a way of feedback regulation**

1. Overexpression of C-JUN suppresses the mRNA level of ACSL4 in B16-F10 cells. RT-qPCR analysis of ACSL4 expression is shown. (B) Overexpression of ELK1 suppresses the mRNA level of ACSL4 in B16-F10 cells. RT-qPCR analysis of ACSL4 expression is shown. (C) Overexpression of HSF1 suppresses the mRNA level of ACSL4 in B16-F10 cells. RT-qPCR analysis of ACSL4 expression is shown. (D) Overexpression of STAT3 suppresses the mRNA level of ACSL4 in B16-F10 cells. RT-qPCR analysis of ACSL4 expression is shown. (E) Knockdown of C-JUN promotes the mRNA level of ACSL4 in B16-F10 cells. RT-qPCR analysis of ACSL4 expression is shown. (F) Knockdown of ELK1 promotes the mRNA level of ACSL4 in B16-F10 cells. RT-qPCR analysis of ACSL4 expression is shown. (G) Knockdown of HSF1 promotes the mRNA level of ACSL4 in B16-F10 cells. RT-qPCR analysis of ACSL4 expression is shown. (H) HSF1 activator HSF1A (10 μM for 12h) treatment suppresses ACSL4 expression in B16-F10 cells. Western blot images confirm the expression of the indicated protein. (I) HSF1 activator HSF1A (10 μM for 12h) treatment suppresses the mRNA level of ACSL4 in B16-F10 cells. RT-qPCR analysis of ACSL4 expression is shown. (J) HSF1 inhibitor DTHIB (10 μM for 12h) treatment promotes ACSL4 expression in B16-F10 cells. Western blot images confirm the expression of the indicated protein. (K) HSF1 inhibitor DTHIB (10 μM for 12h) treatment promotes the mRNA level of ACSL4 in B16-F10 cells. RT-qPCR analysis of ACSL4 expression is shown. (L) STAT3 inhibitor STAT3-IN-1 (10 μM for 12h) treatment promotes ACSL4 expression in B16-F10 cells. Western blot images confirm the expression of the indicated protein. (M) STAT3 inhibitor STAT3-IN-1 (10 μM for 12h) treatment promotes the mRNA level of ACSL4 in B16-F10 cells. RT-qPCR analysis of ACSL4 expression is shown. (N) Knockdown of C-JUN rescues the expression of ACSL4 in FAK KD or SRC KD B16-F10 cells. Western blot images confirm the expression of the indicated protein. (O) Knockdown of ELK1 rescues the expression of ACSL4 in FAK KD or SRC KD B16-F10 cells. Western blot images confirm the expression of the indicated protein. (P) Knockdown of HSF1 rescues the expression of ACSL4 in FAK KD or SRC KD B16-F10 cells. Western blot images confirm the expression of the indicated protein. (Q) Inhibition of STAT3 by STAT3-IN-1 (10 μM for 12h) rescues the expression of ACSL4 in FAK KD or SRC KD B16-F10 cells. Western blot images confirm the expression of the indicated protein. (R) Overexpression of C-JUN partially suppresses the elevated expression of ACSL4 in FAK OE or SRC OE cells. Western blot images confirm the expression of the indicated protein. (S) Overexpression of ELK1 partially suppresses the elevated expression of ACSL4 in FAK OE or SRC OE cells. Western blot images confirm the expression of the indicated protein. (T) Overexpression of HSF1 partially suppresses the elevated expression of ACSL4 in FAK OE or SRC OE cells. Western blot images confirm the expression of the indicated protein. (U) Overexpression of STAT3 partially suppresses the elevated expression of ACSL4 in FAK OE or SRC OE cells. Western blot images confirm the expression of the indicated protein. (V) Analysis of binding kinetics of activators (ATF2, NFATC1, NFATC3, and SMAD4) vs. repressors (c-JUN, ELK1 and HSF1) with ChIP assay. ChIP was performed using indicated antibodes or control IgG. Values are percentage of input.  Enrichment was normalized to the IgG control. Relative binding induced by erastin treatment was normalized to DMSO control.

All data are mean ± SD. from n = 3 biological replicates. **P < 0.05, **P < 0.01, ***P < 0.001, ****P < 0.0001* by two-tailed t-test.

**Figure S7. Oncogenic FAK/SRC-JNK Axis promotes cancer cells ferroptosis *in vivo***

(A)The elevated expression of FAK, SRC or JNK1 in cancer tissues compared to adjacent normal tissues. BRCA (Breast invasive carcinoma), COAD (Colon adenocarcinoma), LGG (Brain Lower Grade Glioma), PAAD (Pancreatic adenocarcinoma), SARC (Sarcoma), STAD (Stomach adenocarcinoma), THYM (Thymoma), ESCA (Esophageal carcinoma), GBM (Glioblastoma multiforme), KIRP (Kidney renal papillary cell carcinoma), LIHC (Liver hepatocellular carcinoma), LUAD (Lung adenocarcinoma), LUSC (Lung squamous cell carcinoma), PCPG (Pheochromocytoma and Paraganglioma). (B) High expression of FAK, SRC or JNK1 is corelated with poor overall survial in several types of cancer patients. THYM (Thymoma), BRCA (Breast invasive carcinoma), LIHC (Liver hepatocellular carcinoma), SARC (Sarcoma).
