# Supplementary figures and images for: FAK/SRC-JNK axis promotes ferroptosis via upregulating ACSL4 expression
Source: Cell Death Dis. 2026 Mar 20;17(1):328. doi: 10.1038/s41419-026-08570-y (PMC13039192; doi:10.1038/s41419-026-08570-y)

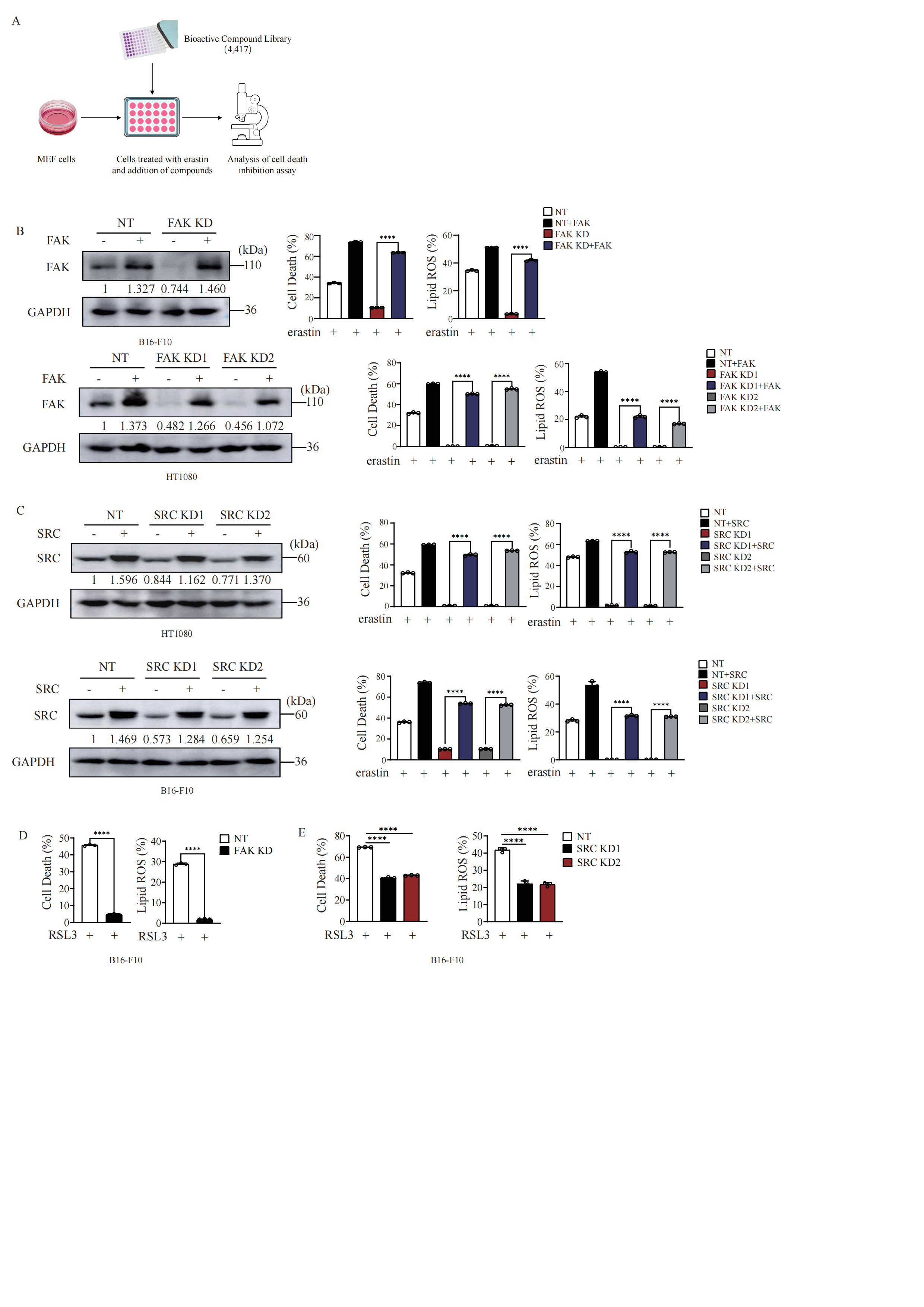

Supplement: Supplementary file 2 — Supplementary figure 1 [file 41419_2026_8570_MOESM2_ESM.tif]

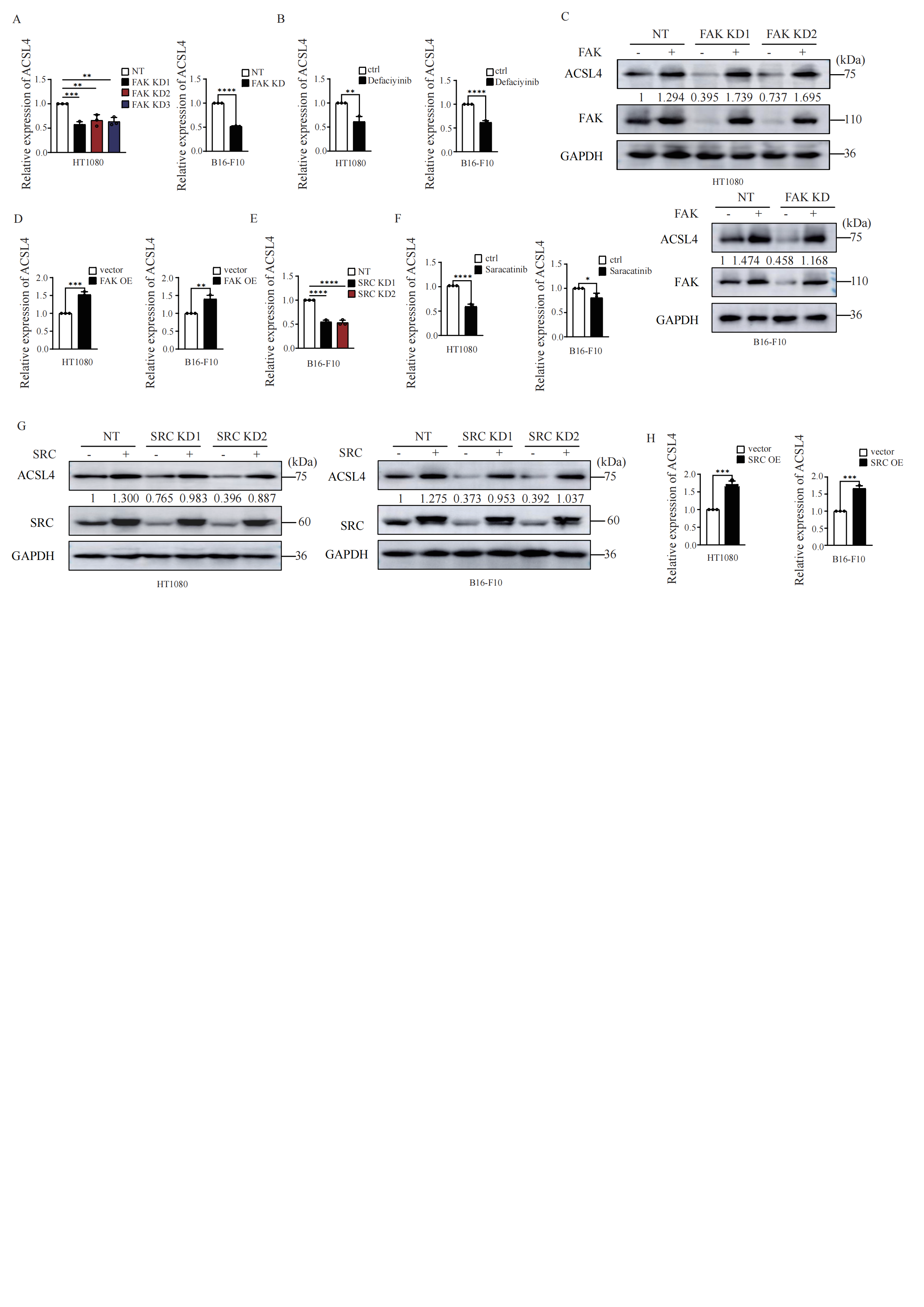

Supplement: Supplementary file 3 — Supplementary figure 2 [file 41419_2026_8570_MOESM3_ESM.tif]

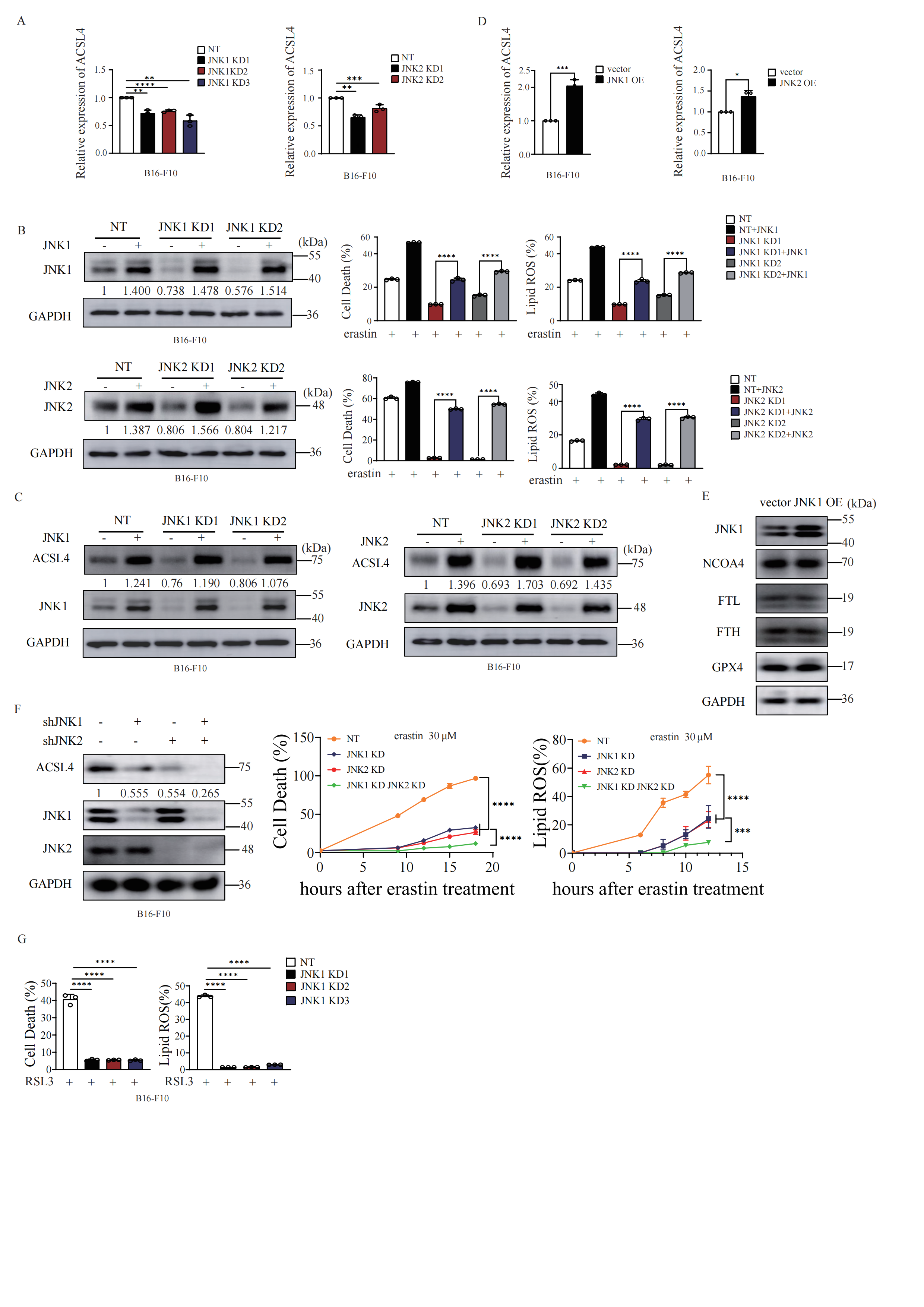

Supplement: Supplementary file 4 — Supplementary figure 3 [file 41419_2026_8570_MOESM4_ESM.tif]

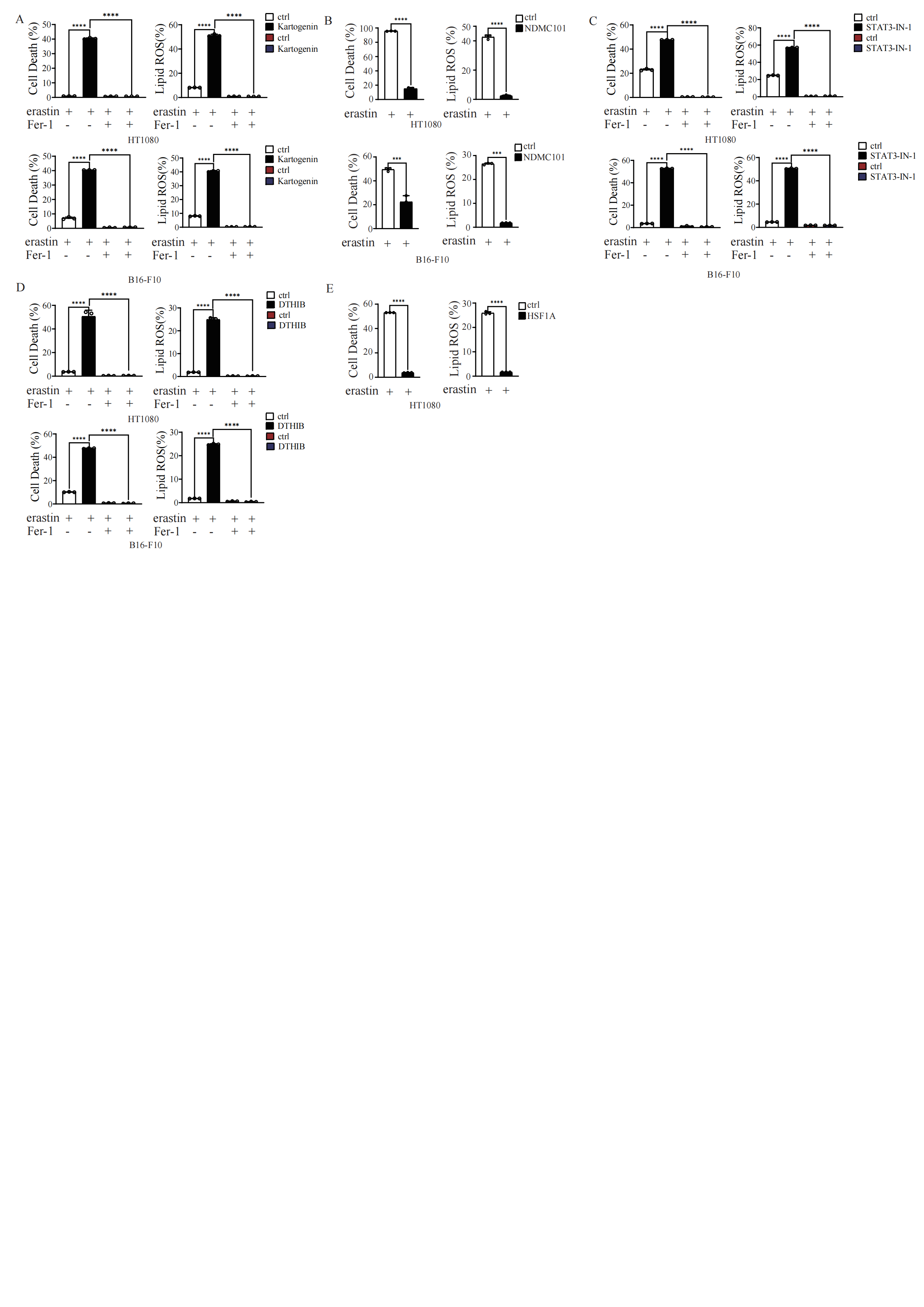

Supplement: Supplementary file 5 — Supplementary figure 4 [file 41419_2026_8570_MOESM5_ESM.tif]

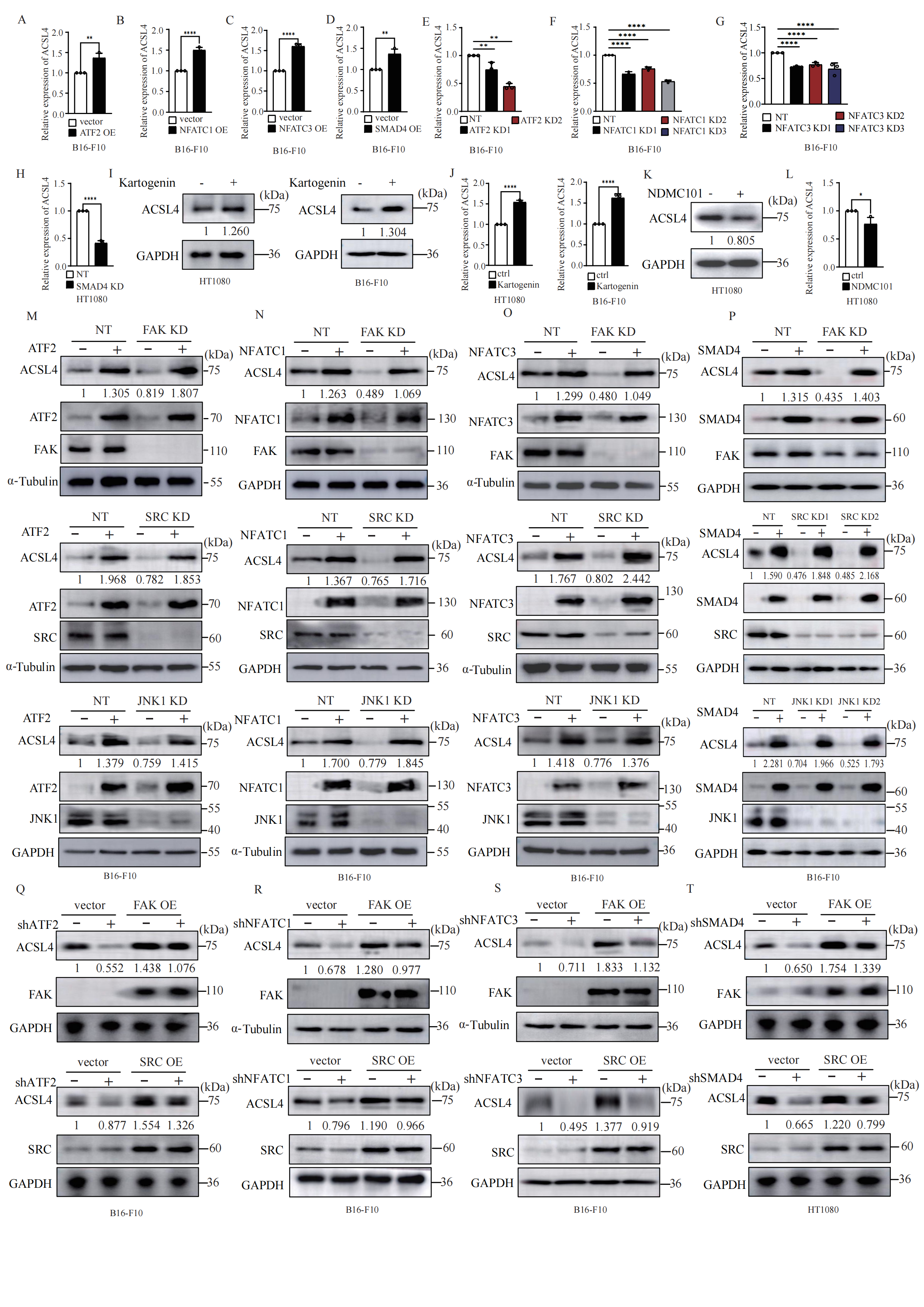

Supplement: Supplementary file 6 — Supplementary figure 5 [file 41419_2026_8570_MOESM6_ESM.tif]

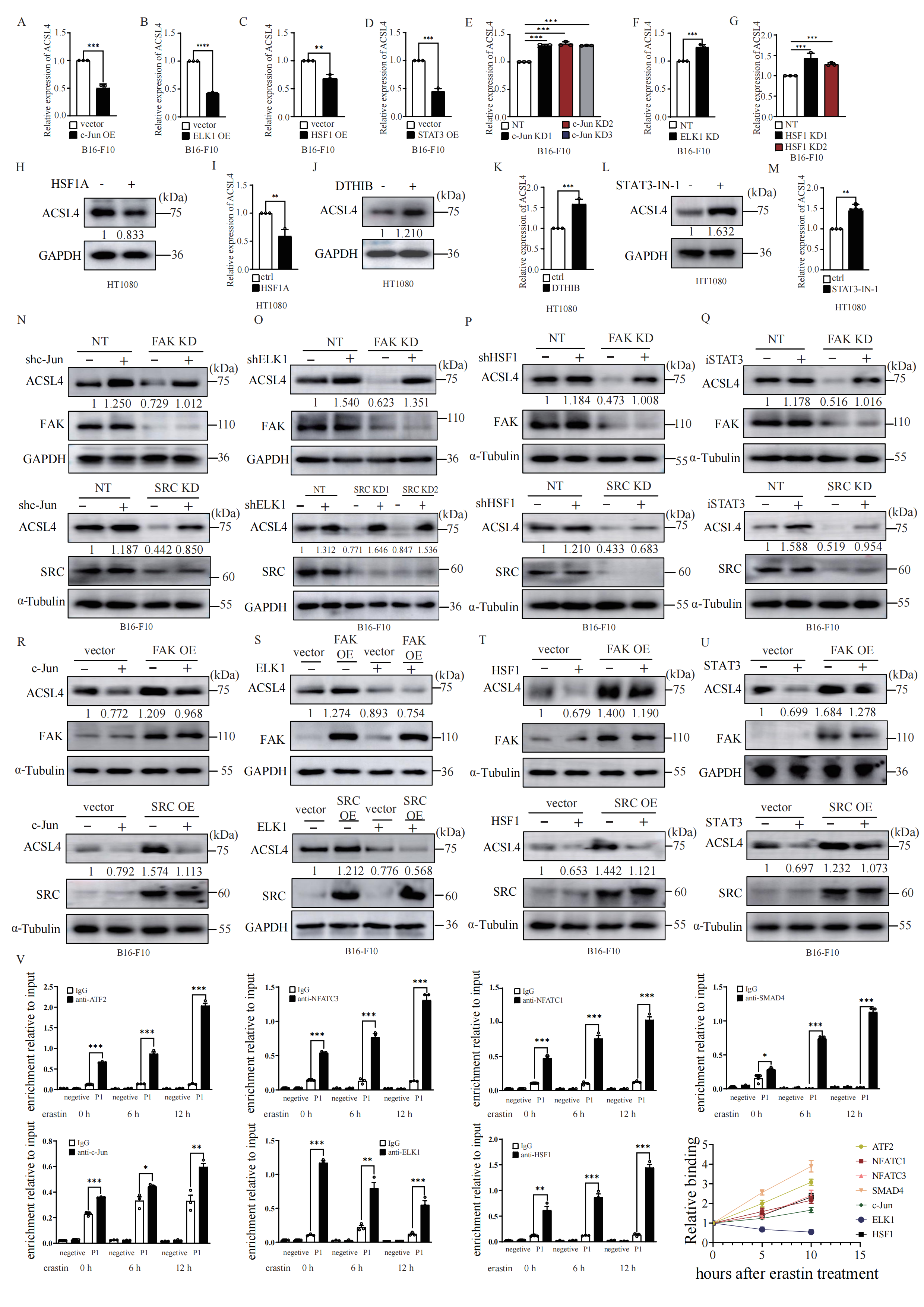

Supplement: Supplementary file 7 — Supplementary figure 6 [file 41419_2026_8570_MOESM7_ESM.tif]

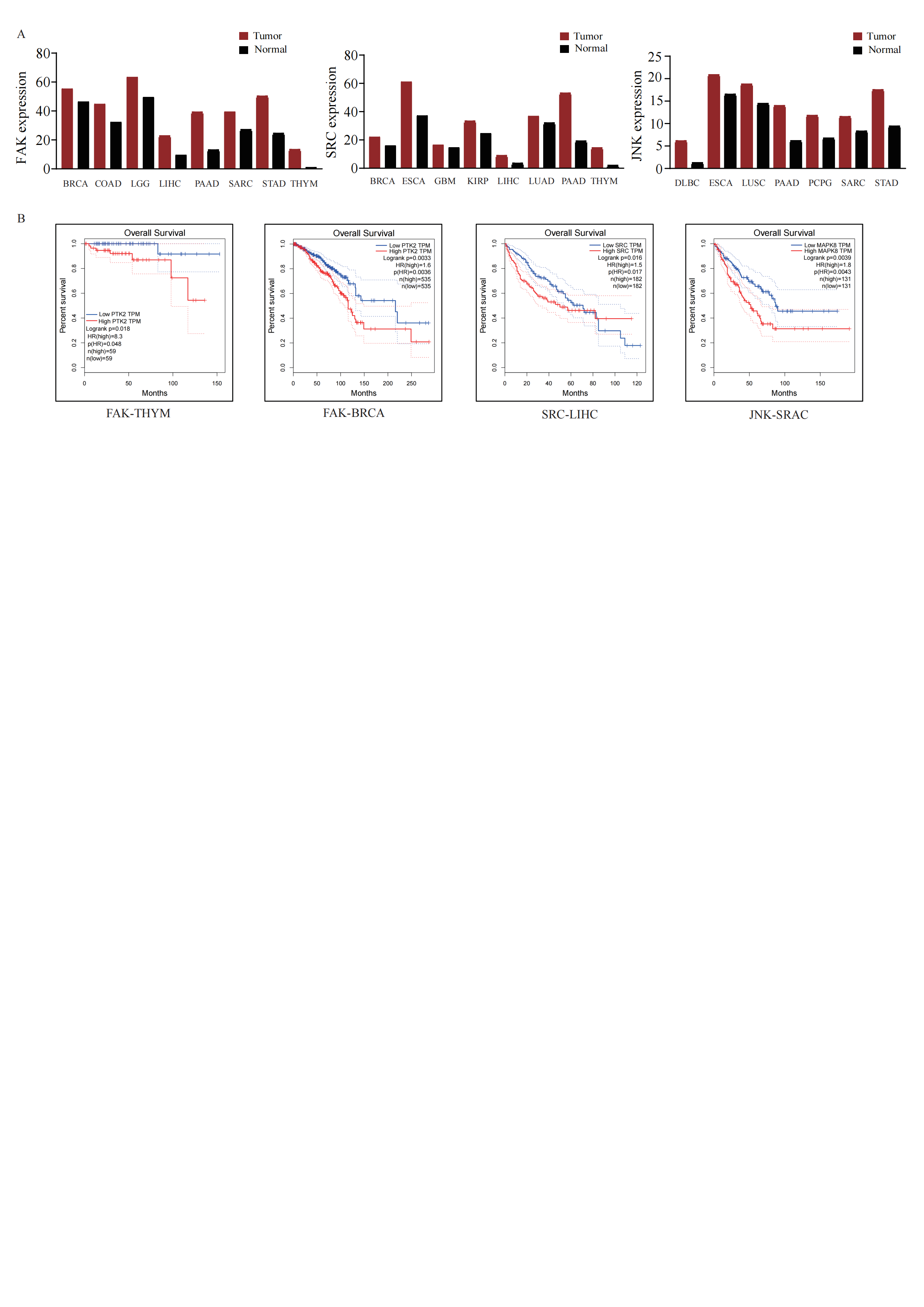

Supplement: Supplementary file 8 — Supplementary figure 7 [file 41419_2026_8570_MOESM8_ESM.tif]

**Fig 1**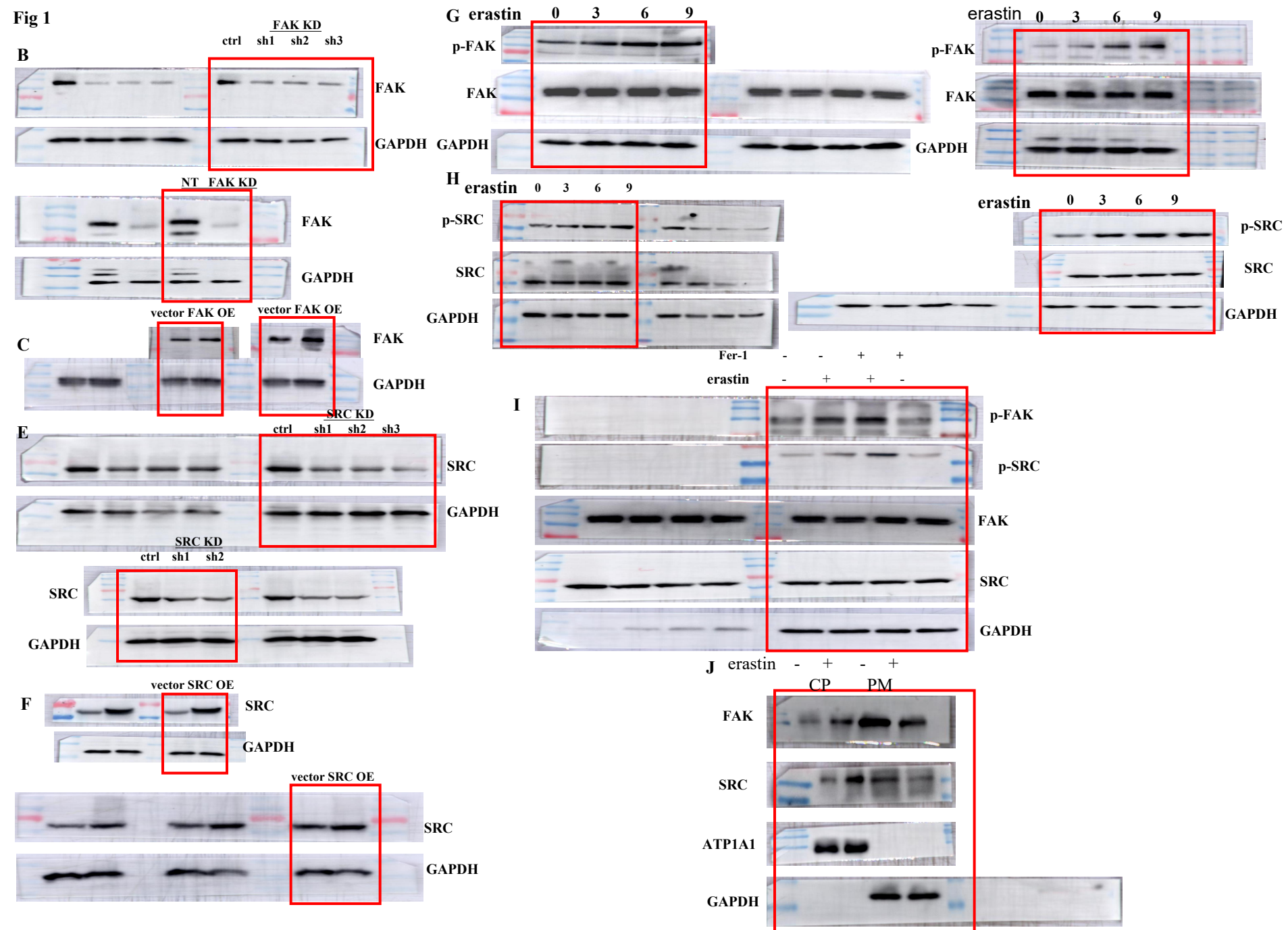

**Fig 2**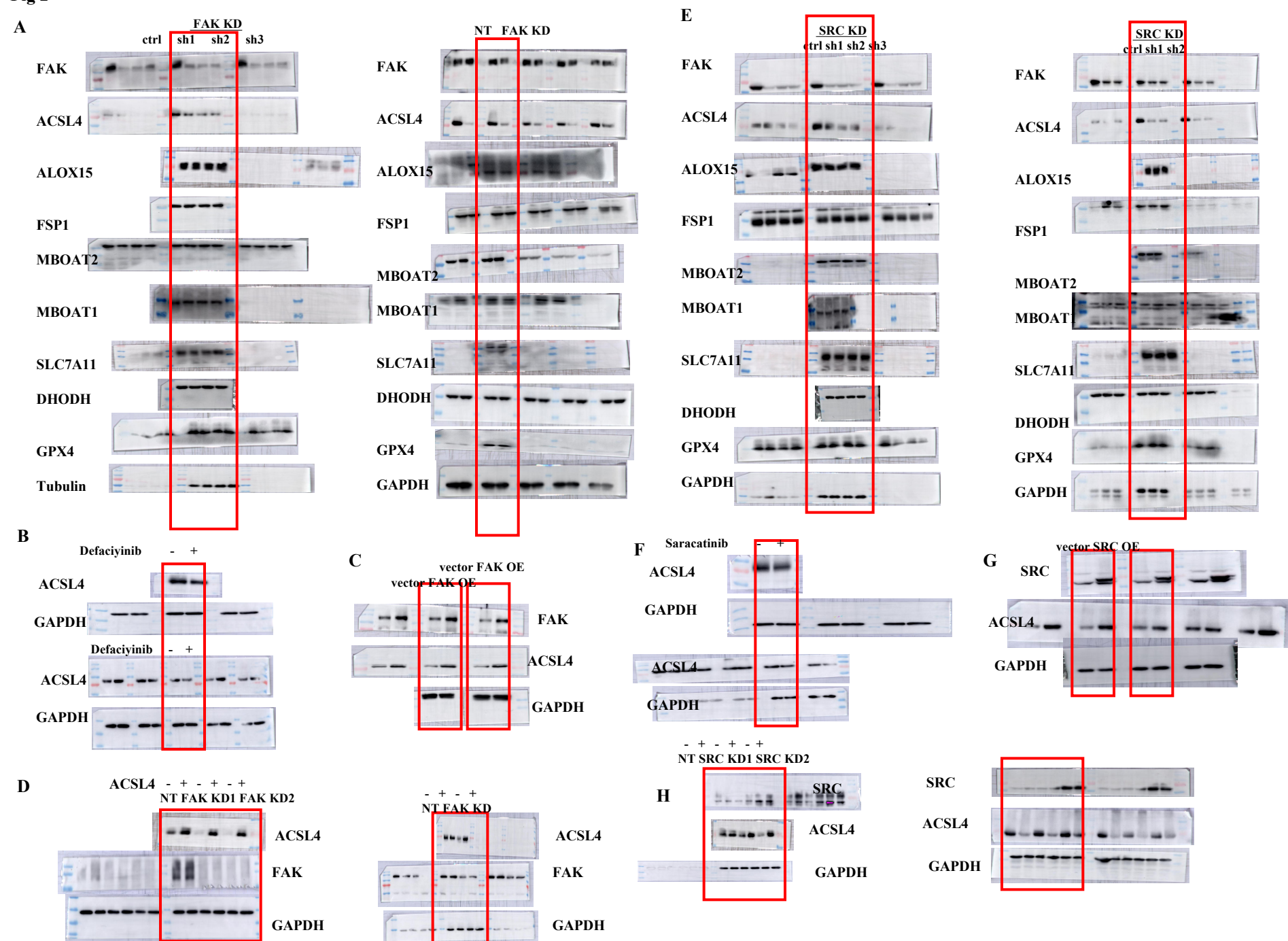

Fig

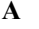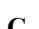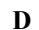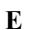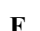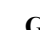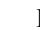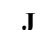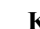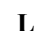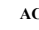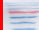

**Fig 4**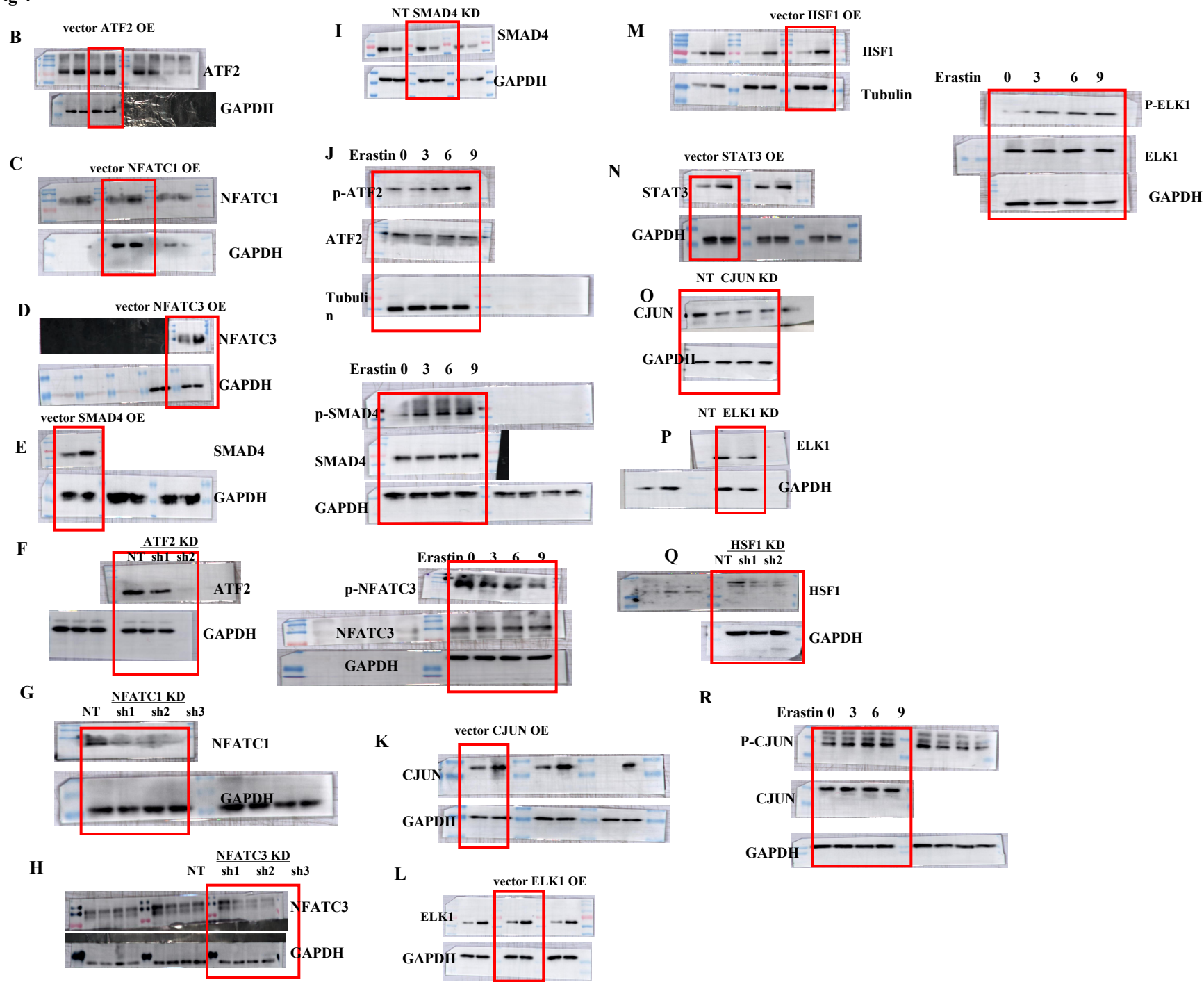

**Fig 5**

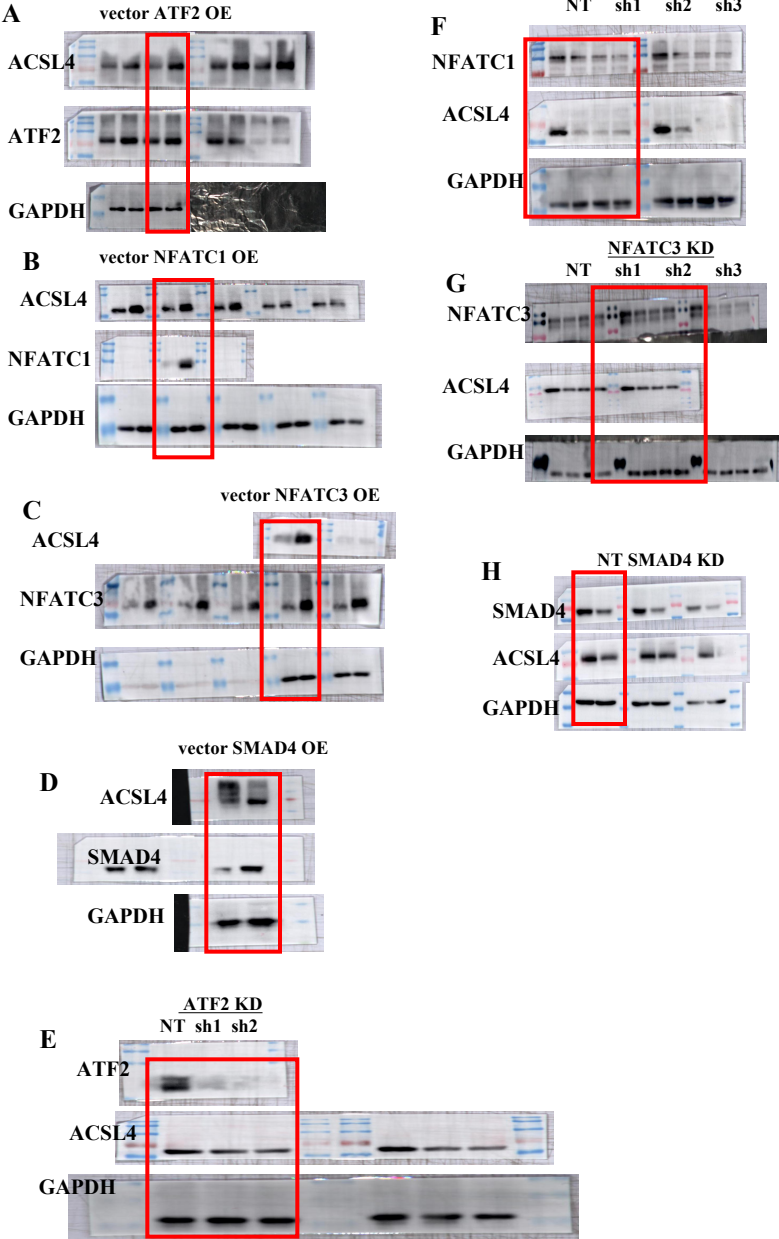

**Fig 6**

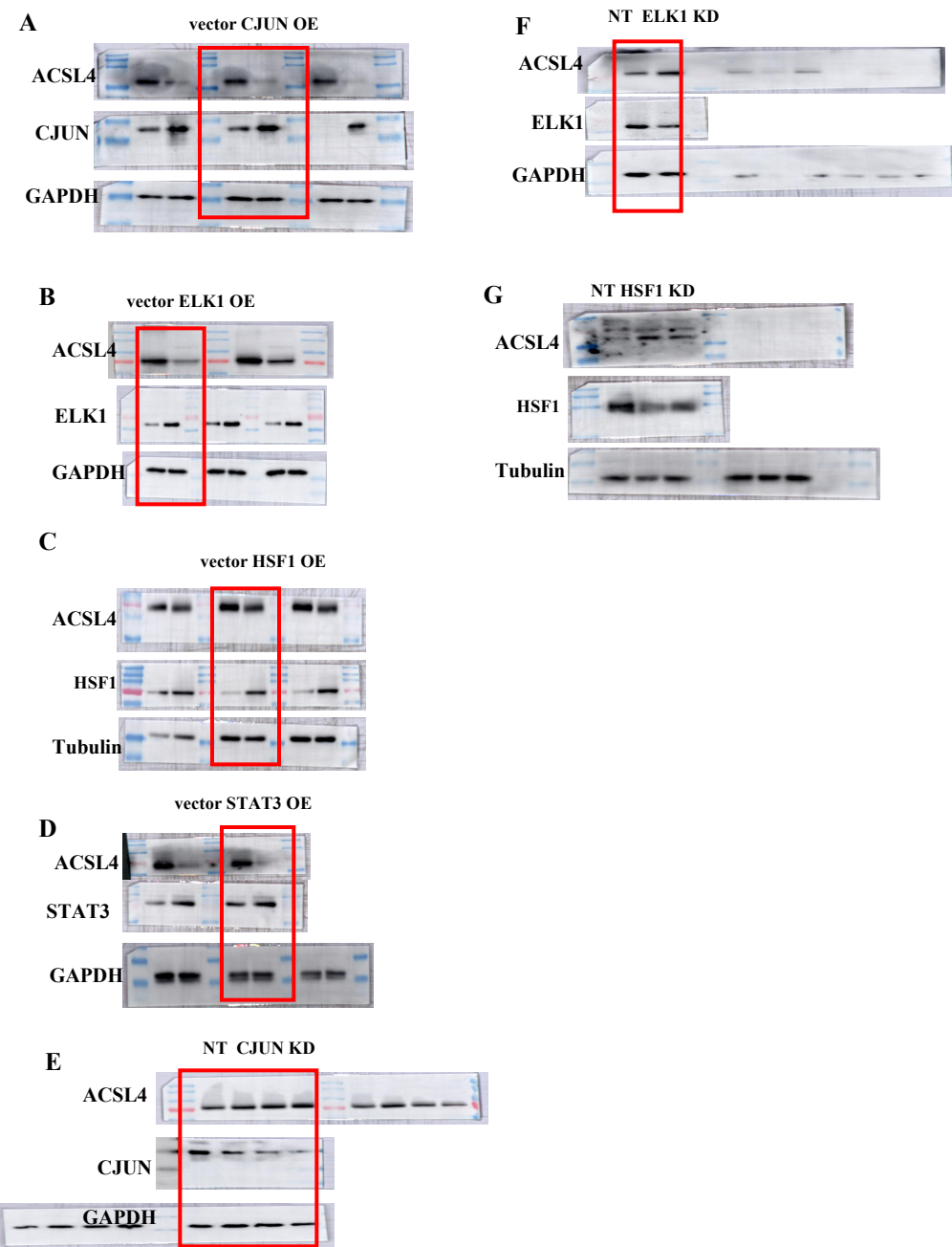

**Fig 6**

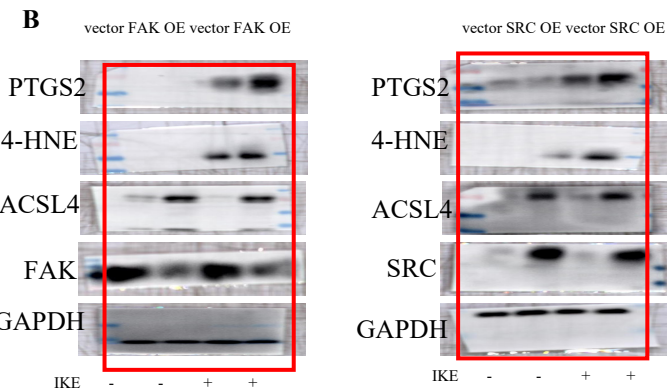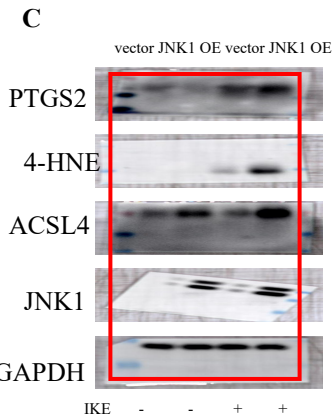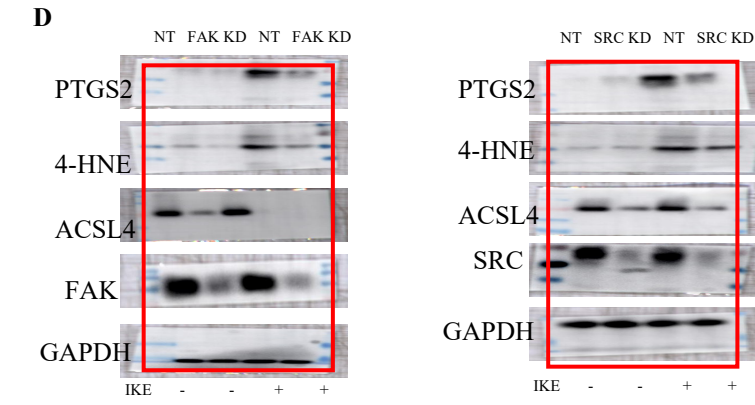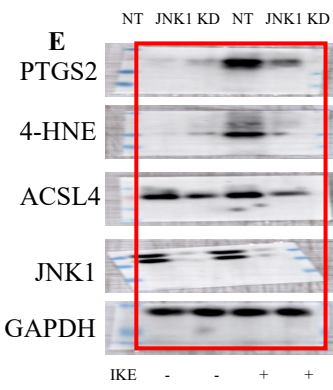

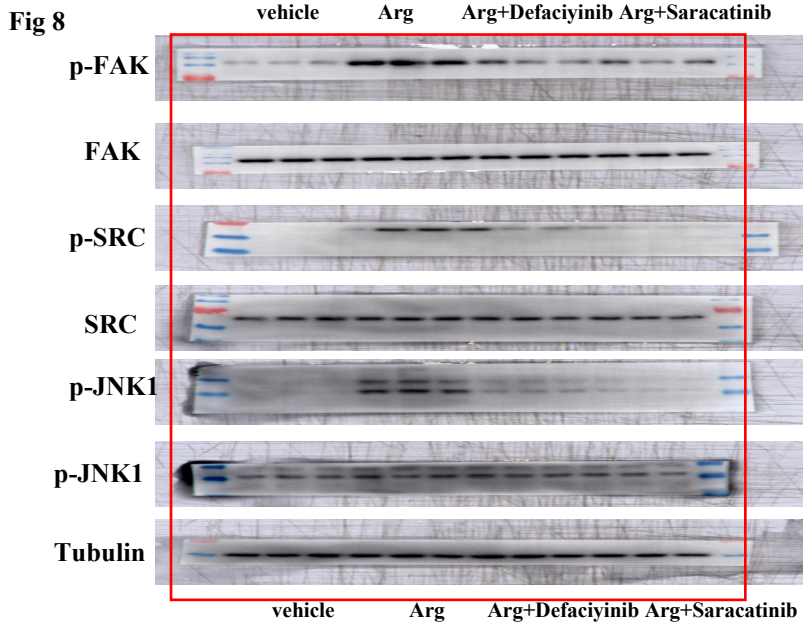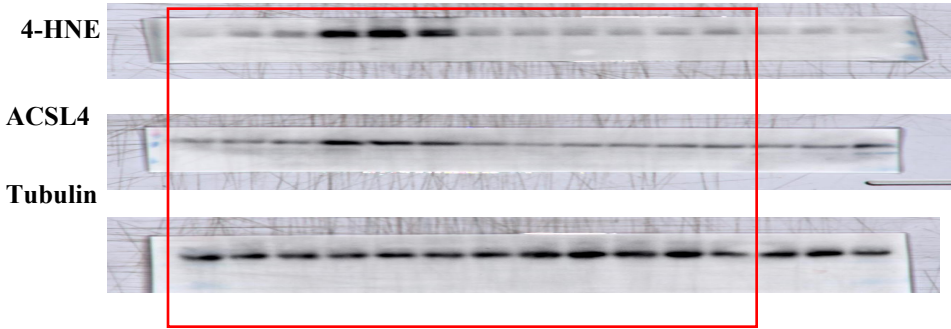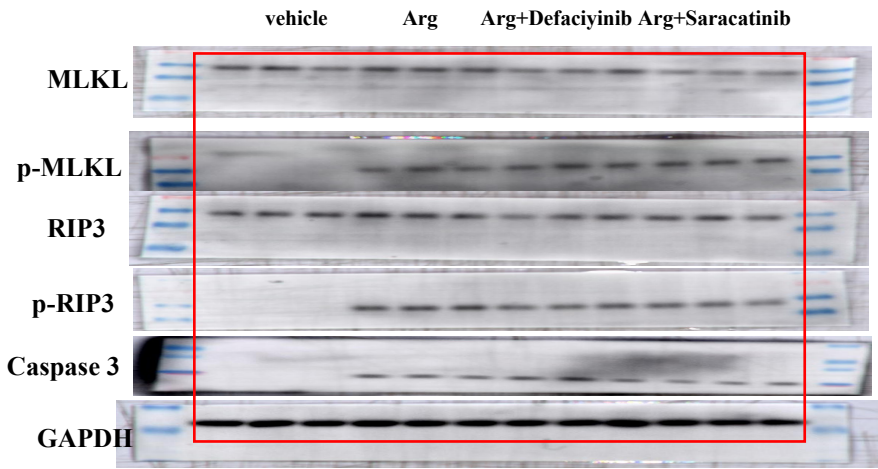

**Fig S1**

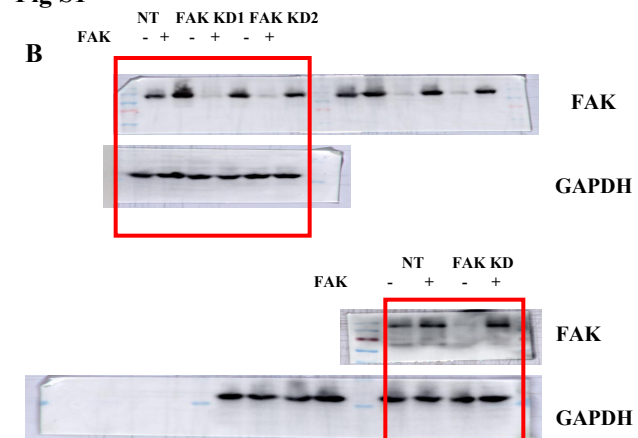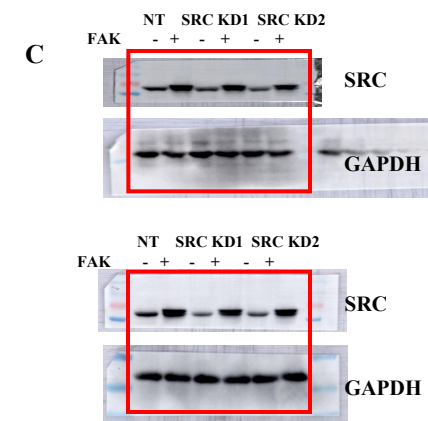

Fig S2

C

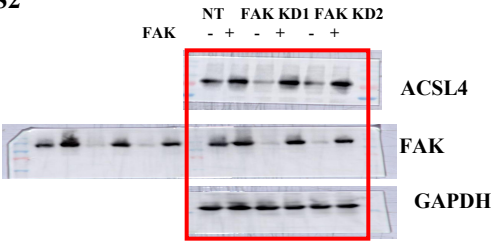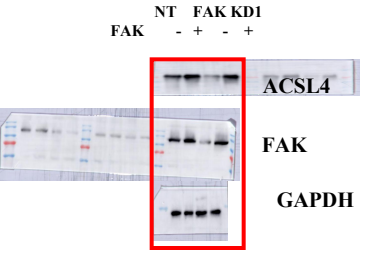

G

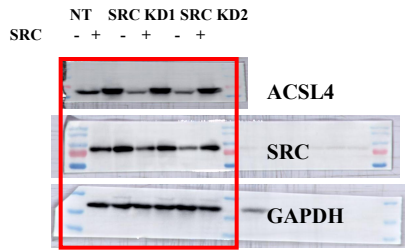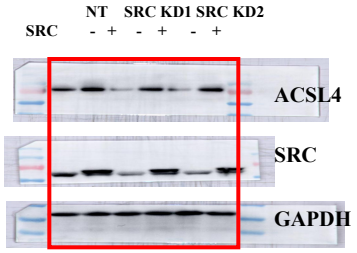

**Fig S3**

**B**

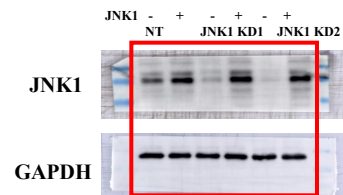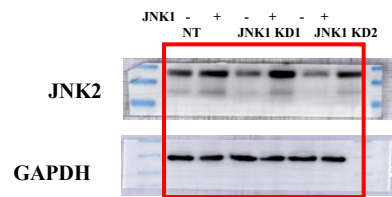

**C**

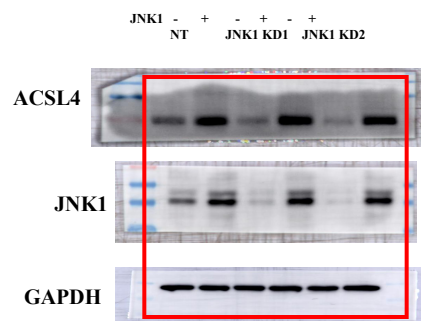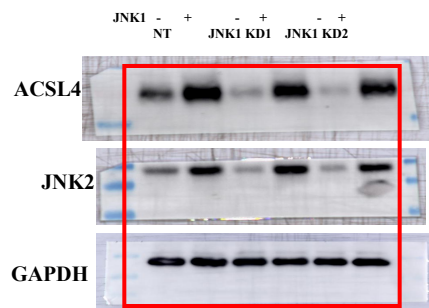

**E**

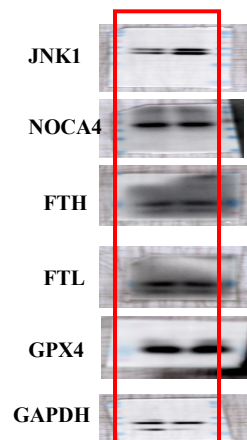

shJNK1 - + - +

**F**

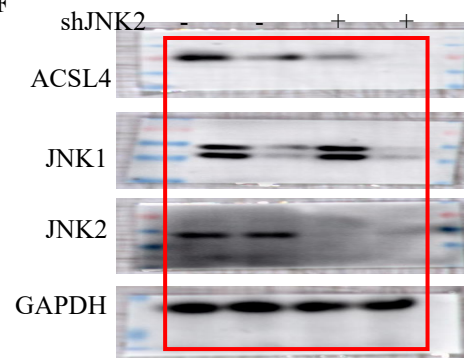

**Fig S5**

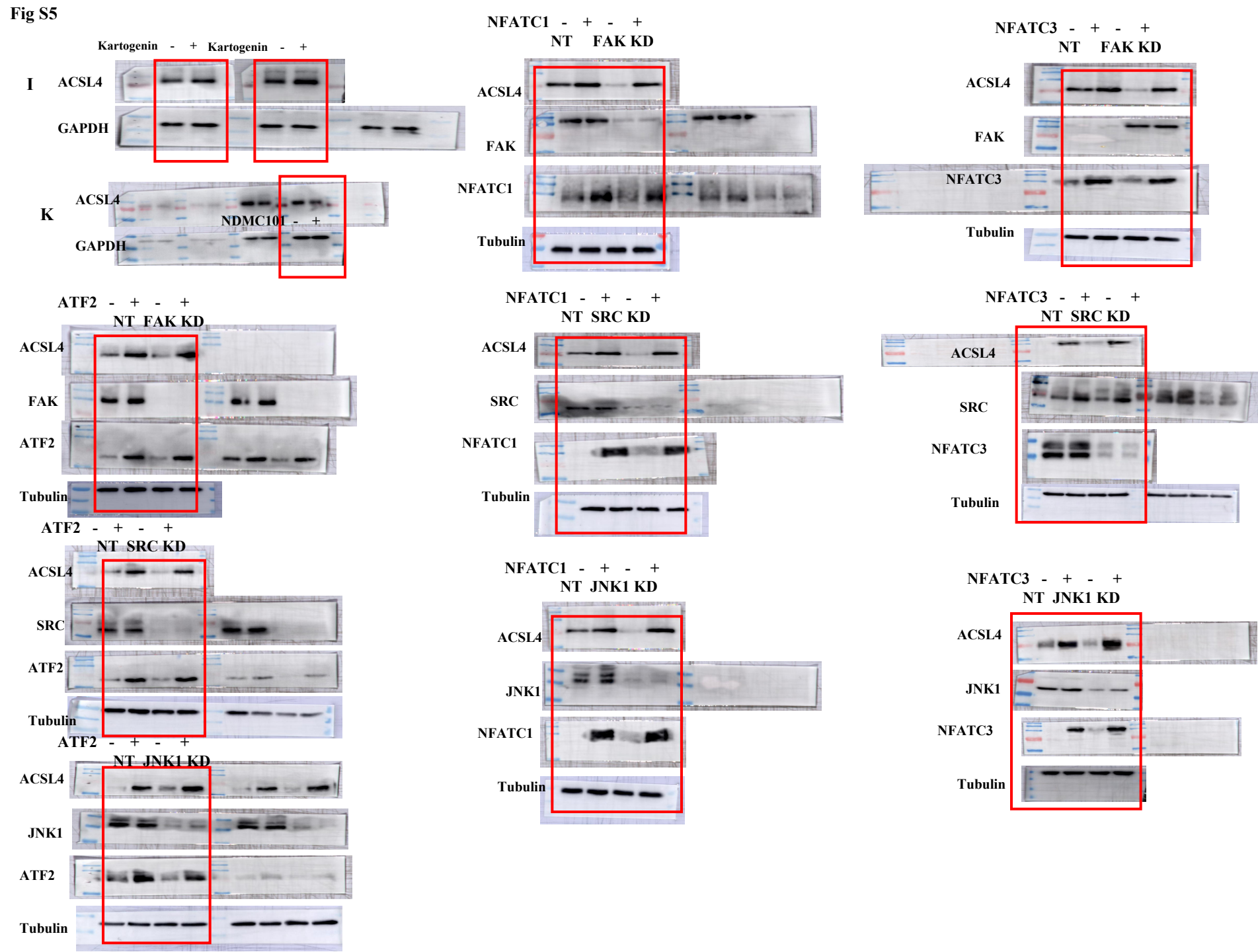

Fig S5

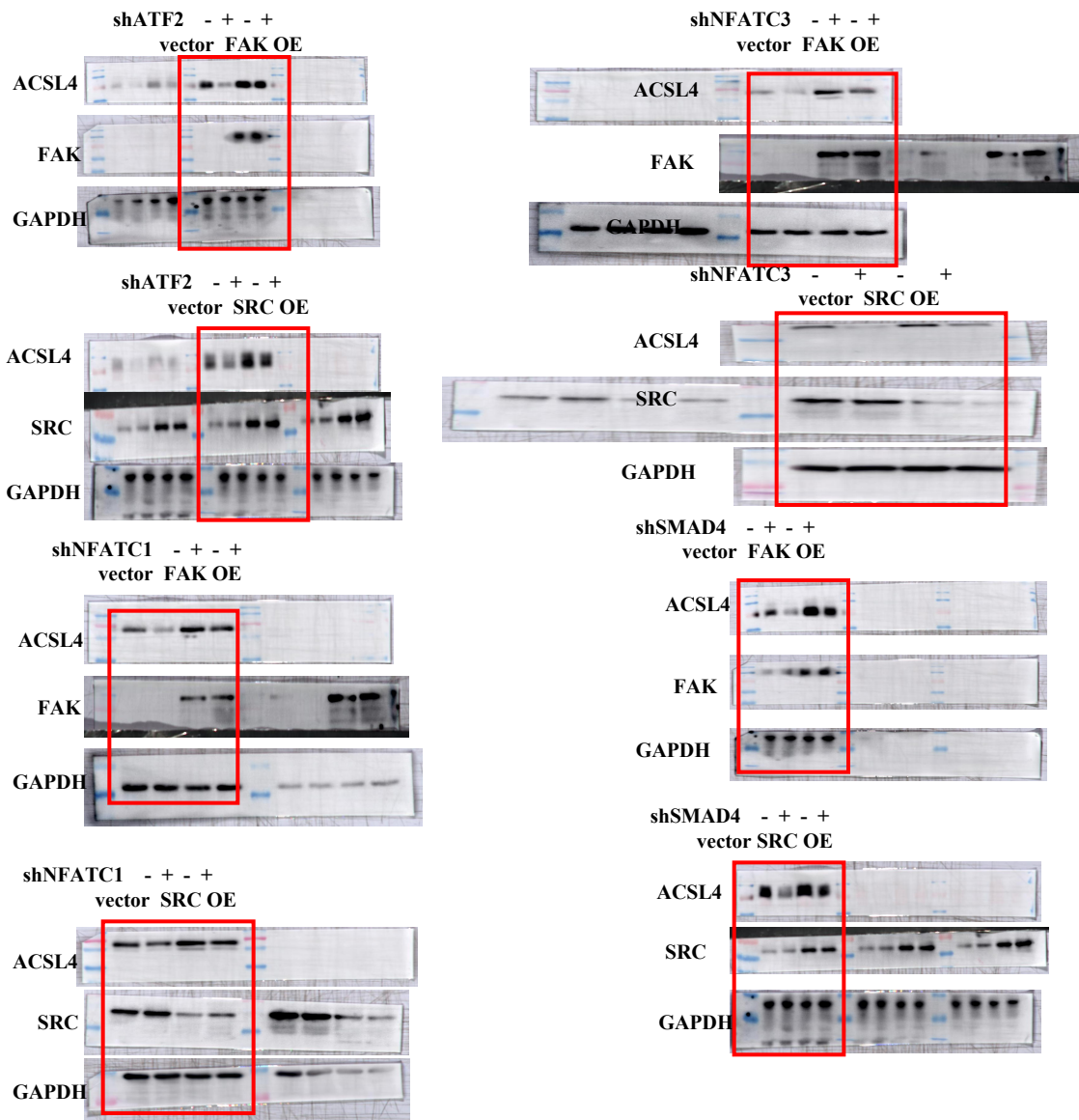

**Fig S6**

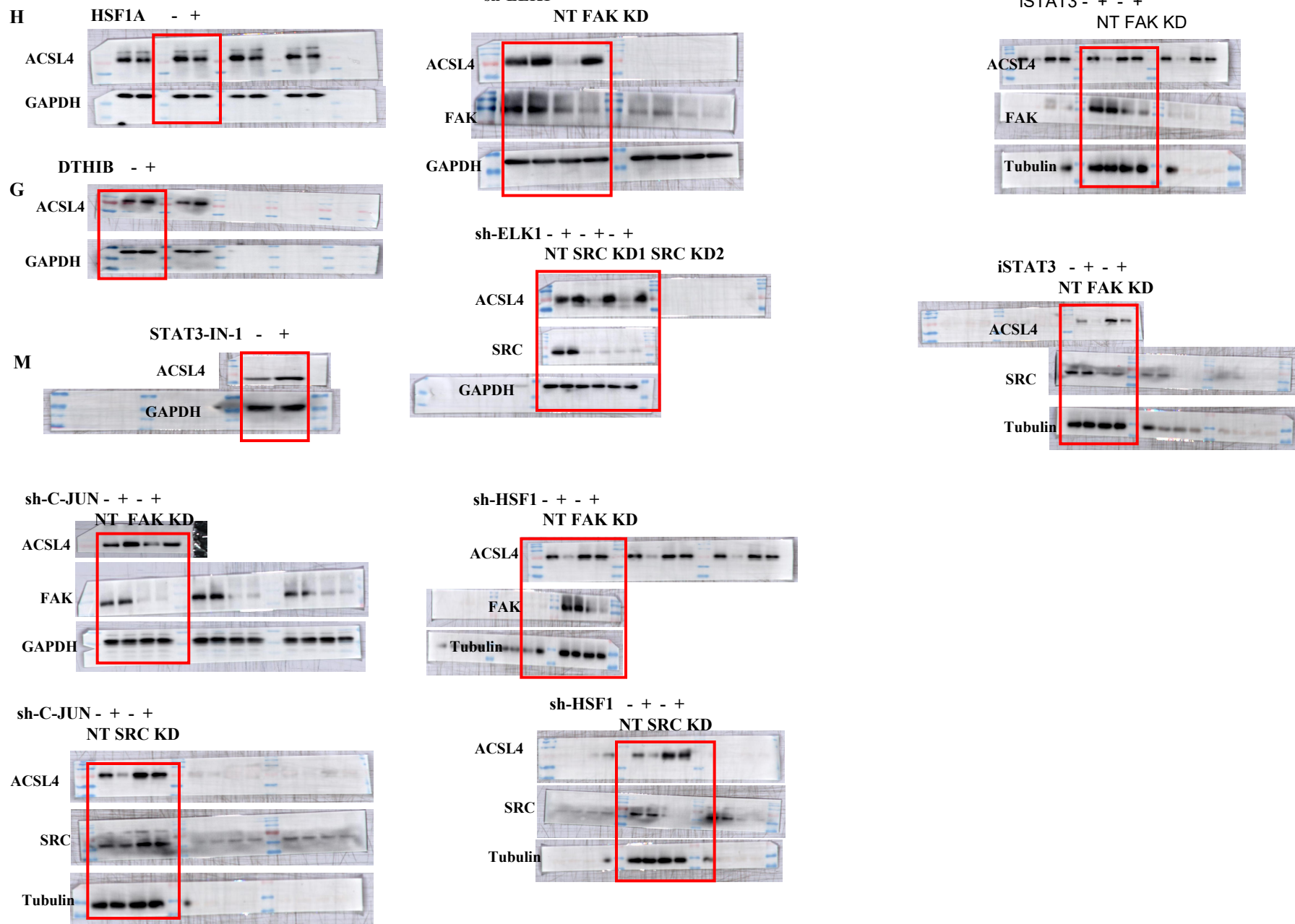

Fig S6

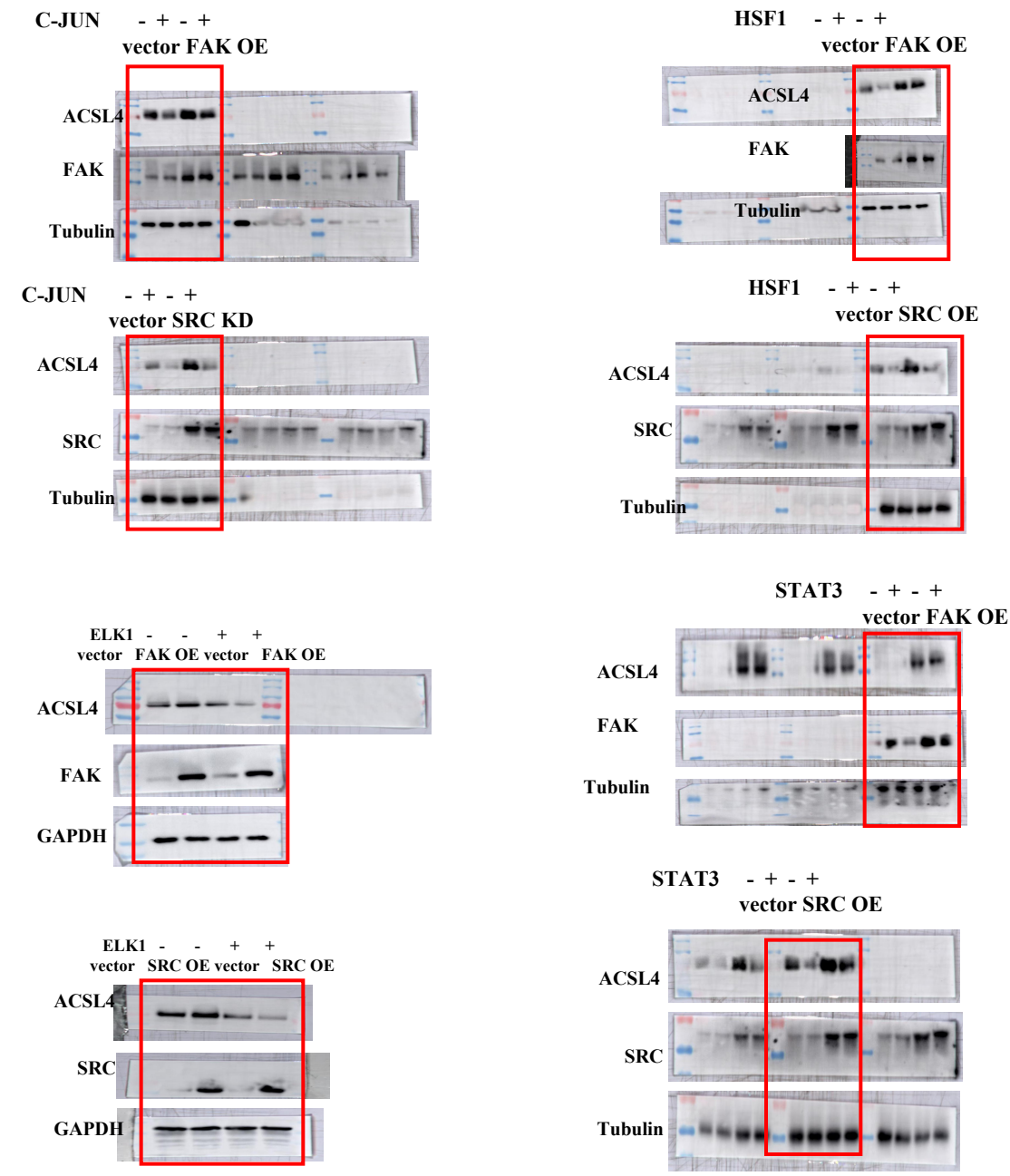

Supplement: Supplementary file 9 — uncropped westen blot image [file 41419_2026_8570_MOESM9_ESM.pdf]
